# Mental health and the overall tendency to follow official recommendations against COVID-19: A U-shaped relationship?

**DOI:** 10.1371/journal.pone.0305833

**Published:** 2024-06-25

**Authors:** Bénédicte Apouey, Rémi Yin, Fabrice Etilé, Alan Piper, Claus Vögele

**Affiliations:** 1 Paris School of Economics (PSE), Paris, France; 2 Centre National de la Recherche Scientifique (CNRS), Paris, France; 3 University of Luxembourg, Esch-sur-Alzette, Luxembourg; 4 UMR 1393 Paris - Jourdan Sciences Économiques, Institut National de Recherche pour l’Agriculture, l’Alimentation et l’Environnement, Paris, France; 5 Economics Department, Leeds University Business School, Leeds, United Kingdom; 6 International Public Economics Department, Freie Universität Berlin, Berlin, Germany; 7 Department of Behavioural and Cognitive Sciences, University of Luxembourg, Esch-sur-Alzette, Luxembourg; University of Glasgow, UNITED KINGDOM

## Abstract

This paper investigates the association between several mental health indicators (depression, anxiety, stress, and loneliness) and the overall tendency to follow official recommendations regarding self-protection against COVID-19 (i.e., overall compliance). We employ panel data from the COME-HERE survey, collected over four waves, on 7,766 individuals (22,878 observations) from France, Germany, Italy, Spain, and Sweden. Employing a flexible specification that allows the association to be non-monotonic, we find a U-shaped relationship, in which transitions to low and high levels of mental health are associated with higher overall compliance, while transitions to medium levels of mental health are associated with less overall compliance. Moreover, anxiety, stress, and loneliness levels at baseline (i.e., at wave 1) also have a U-shaped effect on overall compliance later (i.e., recommendations are followed best by those with lowest and highest levels of anxiety, stress, and loneliness at baseline, while following the recommendations is lowest for those with moderate levels of these variables). These U shapes, which are robust to several specifications, may explain some of the ambiguous results reported in the previous literature. Additionally, we observe a U-shaped association between the mental health indicators and a number of specific health behaviours (including washing hands and mask wearing). Importantly, most of these specific behaviours play a role in overall compliance. Finally, we uncover the role of gender composition effects in some of the results. While variations in depression and stress are negatively associated with variations in overall compliance for men, the association is positive for women. The U-shaped relation in the full sample (composed of males and females) will reflect first the negative slope for males and then the positive slope for females.

## Introduction

Across different countries, governments took strong actions to fight the COVID-19 pandemic, including restrictions surrounding social interactions (e.g., lockdown) and the development of guidelines to slow the spread of the infection [1]. Previous reviews have highlighted the negative consequences of the COVID-19 pandemic (i.e., infections and restrictions) on mental health [2–4].

While health behaviours to protect from infection (e.g., social distancing and hand washing) may influence mental health, mental health may in turn affect health behaviours. It is difficult to say a priori whether mental health will have a positive or negative impact on behaviours. On the one hand, poorer mental health may have a detrimental effect on health behaviours. This is consistent with evidence that anxiety is associated with an increase in willingness to be exposed to the risk of COVID-19 infection [5]. However, on the other hand, we may speculate that individuals in worse mental health (in particular, individuals experiencing more anxiety) may be more likely to overestimate threat, which may improve health behaviours. Consistently, there are indications that mental health problems are associated with perceived severity of COVID-19 [6]. Further, the perceived likelihood of being personally infected predicts engagement in social distancing and hand washing [7]. This is also consistent with evidence that fear of COVID-19 is a predictor of positive behaviour change (e.g., hand hygiene) [8].

Results from empirical studies on the link between mental health and health behaviours during the pandemic are unclear. Some studies find that poorer mental health goes hand in hand with lower engagement in (protective) health behaviours [9, 10]. In contrast, other articles report that poorer mental health is associated with better health behaviours [11, 12].

Methodological variability can produce opposite-sign estimates of the association between mental health and health behaviours, as illustrated by the following two studies, on individuals aged 18 years or older. Zhao et al. find, in a Hongkong sample, a relationship between lower stress levels, and less anxiety and less depressive symptoms (i.e., better mental health) on the one hand, and perceived compliance with social distancing measures on the other [10]. In contrast, Lee et al. report, for another Hongkong sample, a relationship between mental distress (i.e., poorer mental health) and compliance with precautionary measures [13].

The extant empirical literature therefore provides ambiguous evidence about the relationship between mental health and health behaviours. One key issue concerns the question whether this reflects true heterogeneity in the relationship, or whether this stems from limitations of the data and of the econometric strategies employed. Regarding the data, most of the published studies employ cross-sectional data with small samples from a single country (e.g., [13]), with the usual shortcomings: potential for reverse causation, a lack of control for fixed unobserved characteristics, and a lack of statistical power. Moreover, the samples may not be representative of the populations of interest (e.g., [14]). In addition, published articles generally focus on a limited number of health behaviour variables (e.g., [15]), while the relationship between mental health and health behaviours may vary from one behaviour variable to another.

Furthermore, previous papers use econometric specifications that restrict the relationship between mental health and health behaviour to be monotonic (e.g., [10]). This is especially the case when the self-reported scores measuring depression, anxiety, or stress are dichotomised. Dichotomisation does not allow for non-linear relationships, and may produce spuriously opposite results reflecting sample composition effects. Using a more refined measure of mental health, i.e., not just classifying people as having depression symptoms or not, but instead allowing for different levels of depression (such as mild, moderate, and severe depression), is likely to help identify more precisely the shape of the association between mental health and health behaviours.

Given the ambiguity of the literature, as well the methodological limitations of these studies, our paper presents a new exploration of the relationship between mental health and health behaviours. More precisely, we are interested in the causal effect of mental health on health behaviours. We employ unique secondary data from the COME-HERE (COVID-19, MEntal HEalth, REsilience and Self-Regulation) longitudinal survey, which covers five European countries (France, Germany, Italy, Spain, and Sweden) during the pandemic. These countries were chosen because they differ in the date of the outbreak and intensity of the pandemic, the introduction and extent of lockdown measures, and the public policies put in place. The sample size is substantially larger than in many of the previous studies. The data contain information on self-reported depression, anxiety, stress, and loneliness, that we take as indicators of mental health. The data also include information on health behaviours. Within health behaviours, we distinguish the overall tendency to follow recommendations from specific health behaviours (e.g., hand washing or mask wearing). We assess the overall tendency to follow recommendations (which is synonymous with “overall compliance”) by asking individuals a general question on whether they “follow official recommendations” without detailing these recommendations. Our paper addresses several questions: is there an association between mental health and overall compliance? If so, what shape does it have? What is the role of specific health behaviours in the relationship between mental health and overall compliance? To examine the relationship between mental health and health behaviours, we consider different degrees of mental health and use a flexible econometric specification that allows the impact of mental health to be non-monotonic. The longitudinal nature of the data enables us to control for individual heterogeneity and to address reverse causation.

## Method

### Design

Our data come from the pandemic-dedicated COME-HERE (COVID-19, MEntal HEalth, REsilience and Self-Regulation) longitudinal survey, conceived by the University of Luxembourg (see https://pandemic.uni.lu/) and conducted online by Qualtrics. Qualtrics used stratified sampling, and each national sample at wave 1 is representative in terms of gender, region, and age. Ethical approval was obtained from the Ethics Review Panel of the University of Luxembourg (ERP 20–026 C/A). In this panel survey, a representative sample of individuals from five European countries (France, Germany, Italy, Spain, and Sweden) is repeatedly asked questions about psychological health and health behaviours during the pandemic. Data also contain information on demographic factors and socioeconomic status. Our study focuses on waves 1 (April 2020), 2 (June 2020), 3 (August 2020), and 4 (November and December 2020). Our sample of interest contains 7,766 individuals, corresponding to 22,878 observations.

### Health behaviours

We assess health behaviours using a variable capturing the overall tendency to follow official recommendations, a series of indicators capturing *specific* health behaviours (the Coronavirus Behavior Scale (CBS) and the Adherence scale (ADH) *items*), and general measures equal to the sum of several specific health behaviours (the CBS and ADH *scores*). All health behaviour measures in our data are self-reported.

Background information on differences in official recommendations in the five countries over time are provided in S1 File.

#### Overall compliance—Overall tendency to follow official recommendations

**Fig 1:**
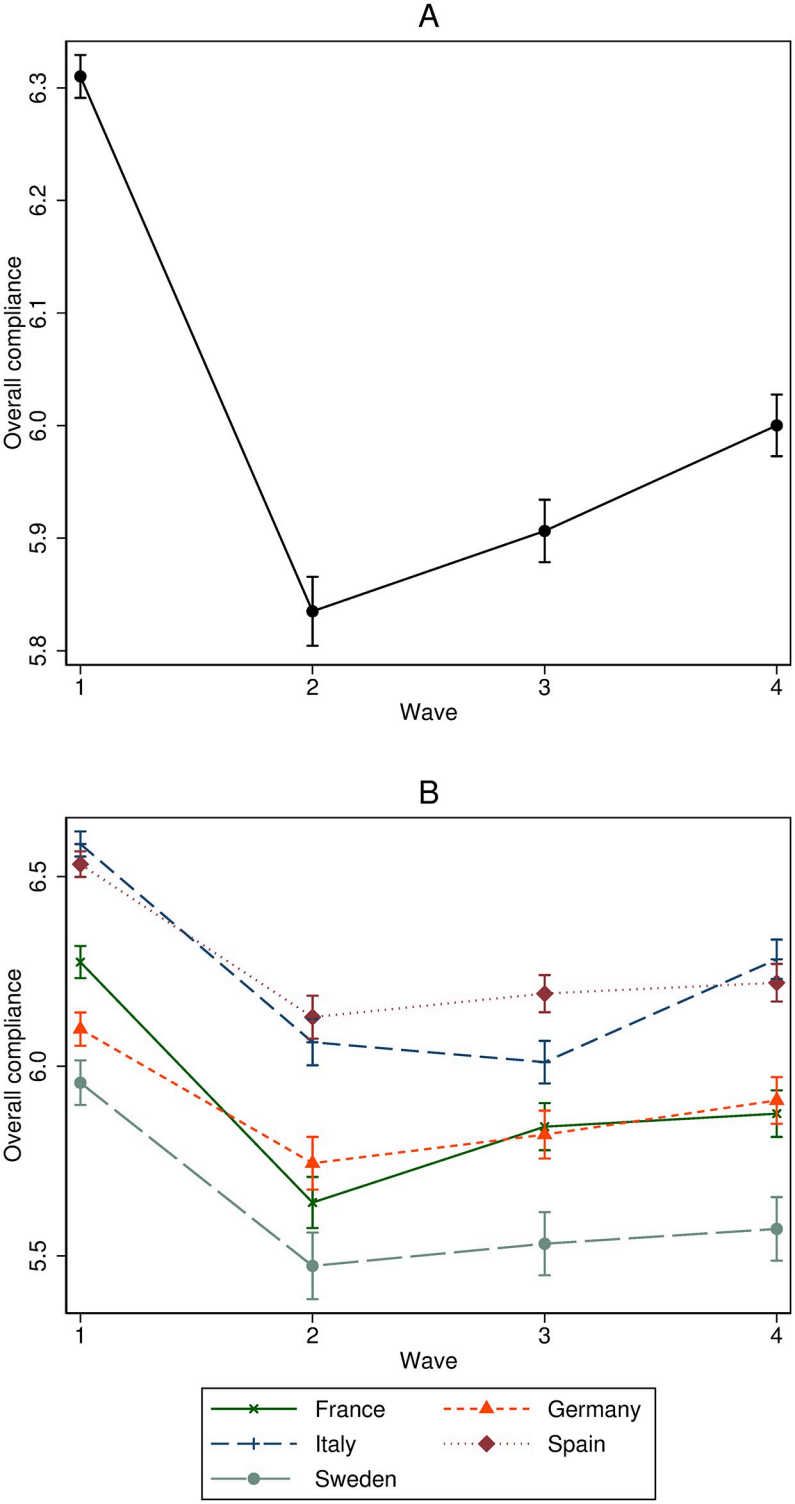
Evolution of following recommendations over time. A: Evolution for all countries. B: Evolution by country.

Our main outcome of interest is overall compliance that is captured by an indicator for following official recommendations overall. In all waves, respondents are asked whether they are “following the recommendations from authorities to prevent the spread of COVID-19,” on a scale running from 1 (“Not at all”) to 7 (“Absolutely”). The mean of the variable equals 6.08 and its standard deviation is 1.19 (for 22,878 observations). Fig 1 shows that, in the full sample, overall compliance drops between April (wave 1) and June 2020 (wave 2), and then steadily increases until the end of 2020 (wave 4). Despite this increase, overall compliance in wave 4 remains lower than in wave 1. Moreover, overall compliance is higher in Italy and Spain, medium in France and Germany, and lower in Sweden.

#### The Coronavirus Behavior Scale (CBS)

In wave 1 (only), we ask the questions of the CBS that contains a list of 14 reasonable and unreasonable specific behaviours. In this article, we focus on the nine reasonable behaviours. The questions are the following: “How much do you agree or disagree with the following statements? I am planning to/have already…”

- “cleaned and disinfected surfaces in my home more often” (CBS_1)
- “bought medical masks” (CBS_2)
- “stayed home when I felt ill” (CBS_3)
- “started avoiding crowded spaces” (CBS_4)
- “bought disinfectant” (CBS_6)
- “limited my travel plans” (CBS_7)
- “started washing my hands more” (CBS_8)
- “shaken hands with people less” (CBS_9)
- “been doing more work or leisure activities from home” (CBS_14)

Responses are provided on a scale running from 1 (“Strongly disagree”) to 5 (“Strongly agree”). We analyse each of these nine specific behaviours separately. We also use the CBS score (for reasonable behaviours) which is the sum of the nine items. While the CBS is not a standardised questionnaire, but was custom-made in the COME-HERE survey, the measure of internal consistency (Cronbach’s alpha) of the CBS score is high (Table A.2 in S1 File).

Because the CBS questions are only asked in the first wave of the COME-HERE data, we cannot estimate models that include individual fixed effects or lagged mental health to study these indicators.

#### The Adherence scale (ADH)

In waves 3 and 4, respondents are asked whether they adopt 16 specific behaviours: “In the past week, how often did you do any of the following?”

- Kept a distance of 2 meters to other people (ADH_1)
- Wore a mask, when the distance of 2 meters could not be kept (ADH_2)
- Avoided shaking hands with or kissing other people (ADH_3)
- Avoided close contact with sick people (ADH_4)
- Washed or disinfected my hands regularly (ADH_5)
- Coughed and sneezed into the crease of my elbow or a tissue (ADH_6)
- Were particularly careful around vulnerable persons (ADH_7)
- Called rather than visited a doctor in case of signs of infection (ADH_8)
- Avoided touching my face (ADH_9)
- Stayed at home, when I felt sick (ADH_10)
- Avoided going out for not immediately necessary activities (ADH_11)
- Avoided unnecessary social contacts (ADH_12)
- Kept a list of people I had close contact with (ADH_13)
- Wore a mask in public transport, shops, and similar public places (ADH_14)
- Wore my disposable mask only once and for max. 8 hours (ADH_15)
- Washed my reusable mask after every day of use at 60°C (ADH_16)

Answers are coded on a scale running from 0 (“Never”) to 5 (“All the time”). Respondents can also answer “Not applicable” for each question. As all these items are part of the global recommendations, we treat the “Not applicable” answers as zeros.

We focus on each of these specific behaviours separately. We also employ three general scores that summarise the information. See details on the construction of the scores using exploratory factor analysis in S1 File (Tables A.3 and A.4, Fig A.2, and related comments).

### Mental health

We examine the relationships between health behaviours and four mental health constructs: depression, anxiety, stress, and loneliness. These constructs are assessed using standardised questionnaires that are commonly used in the literature [9, 10]. They capture self-reported mental health. Importantly, self-assessments of health (including mental health) have been shown to predict mortality and longevity (for an overview, see [16]).

#### Depression

In the current study, we assess self-reported severity of depression symptoms using the brief Patience Health Questionnaire 9 (PHQ-9). This is a standardised questionnaire to screen for depression. The PHQ-9 contains nine items asking for the frequency of depressive symptoms individuals felt in the past two weeks. Items are scored on a four-point Likert scale where 0 indicates “Not at all” and 3 indicates “Nearly every day”. The measure of severity of depressive symptoms is calculated by adding up the responses of the nine items, ranging from 0 to 27. We use cutoff scores of 5, 10, 15, and 20 to capture no, mild, moderate, moderately severe, and severe depression, respectively [17].

#### Anxiety

As is the case for depression, the current study does not employ a clinical psychological assessment, but relies on a self-reported measure assessing anxiety symptoms. Specifically, we use the Generalized Anxiety Disorder (GAD-7) score as a measure of the severity of anxiety. The GAD-7 questionnaire consists of seven items assessing the frequency of anxiety-related problems an individual faced over the last two weeks. Items score on a four-point Likert scale where 0 indicates “Not at all” and 3 indicates “Nearly every day”. The measure of severity of anxiety symptoms is the summary score of the seven items, ranging from 0 to 21, where a higher score represents a higher level of anxiety. Following [18], we use the cutoff scores of 5, 10, 15 to capture no, mild, moderate, and severe anxiety, respectively.

#### Stress

We measure stress using the Perceived Stress Scale (PSS), which assesses how different situations can affect one’s feeling and one’s perceived stress. This standardised questionnaire consists of 10 items where people indicate how often they felt or thought a certain way over the past month. Items are scored on a 5-point Likert scale where 0 indicates “Never” and 4 indicates “Very often”. The PSS score is a summary measure of all 10 items, ranging from 0 to 40, with a higher score indicating higher perceived stress. From this continuous measure, cutoff scores of 13 and 26 can be used to capture low, moderate, and high perceived stress respectively [19, 20].

#### Loneliness

We measure loneliness using the UCLA Loneliness Scale (ULS-8), which is a widely used questionnaire to capture the level of perceived loneliness. The measure consists of eight items assessing the frequency of feeling of loneliness over the past two weeks. Items score on a four-point Likert scale where 1 indicates “Never” and 4 “Often”. The ULS-8 score is the summary measure of the eight items, whereby a higher score represents a higher level of loneliness. Cutoff scores of 13, 20, and 25 are used to represent low, normal to moderate, moderate to high, and high levels of loneliness, respectively [21].

Psychometric properties for all mental health questionnaires are reported in Tables A.5 to A.9 in S1 File and descriptive statistics for mental health are shown in Figs A.3 to A.6 in S1 File.

### Controls

We also use individual-level information (gender, age, marital status, household composition, education, working from home, income, income change, confidence in the government and in health services, and knowledge of the disease) and aggregate-level controls (stringency index and number of deaths). Details about these variables are provided in S1 File.

### Statistical analysis

To describe the relationship between mental health and overall compliance, we regress the latter on a set of dummies for each state of mental health, using the best mental state (e.g., “no depression”) as our reference category. By including dummies rather than a continuous score, we allow for non-linear relationships between mental health and overall compliance. We estimate separate models for each of the four mental health variables, because these variables are highly correlated (Table A.10 in S1 File).

The model controls for demographic characteristics, socioeconomic status, confidence in the government and health services, knowledge, stringency, and the number of deaths. We also include country-wave fixed effects, to capture any unobserved characteristic that is fixed at the country-wave level and that may have an effect on individual health behaviour (for instance, any official public health recommendations and any health, healthcare, or economic policy that is country-period specific). Importantly, because we control for variations in the population-level COVID-19 contamination risk and in governmental health recommendations, the estimated associations do not reflect a full mediating effect between COVID-19-related macro shocks and overall compliance. The associations between mental health and overall compliance are identified from variations in mental health that are independent from the macro shocks.

We first estimate a “Pooled OLS” model, in which overall compliance in wave *t* is regressed on mental health measured in the same wave (and controls and country-wave fixed effects). This model is descriptive and shows associations between mental health and overall compliance, rather than the causal effect of mental health on overall compliance. Indeed, in this regression, mental health may be endogenous because of reverse causation (i.e., it is entirely possible that overall compliance influences mental health) and the omission of third common hidden factors (i.e., factors that have an effect on both mental health and overall compliance) even though we include a number of controls and country-wave fixed effects.

To get closer to causal inference, we employ two methods that exploit the longitudinal nature of the COME-HERE data: the “OLS-FE” and the “Lag” models. The “OLS-FE” model is an OLS regression with individual fixed effects to control for individual characteristics that are fixed over time (such as genetic factors). The model thus takes into account some third common hidden factors. This model identifies the impact of individual transitions in mental health on transitions in overall compliance. In the “Lag” model, we estimate OLS regression models of overall compliance measured in waves 2, 3, and 4 as a function of mental health in wave 1. This is informative of the extent to which individual behaviour is predicted by the initial state of mental health. Any significant associations can then be explained either by a long-lasting effect of mental health state or by some unobserved trait that would simultaneously explain initial mental health and overall compliance later. Given that mental health is measured before overall compliance, reverse causation is unlikely in this model.

In all models, standard errors are clustered at the date (day of interview) and country level.

## The U-shaped relationship between mental health and overall compliance

**Fig 2:**
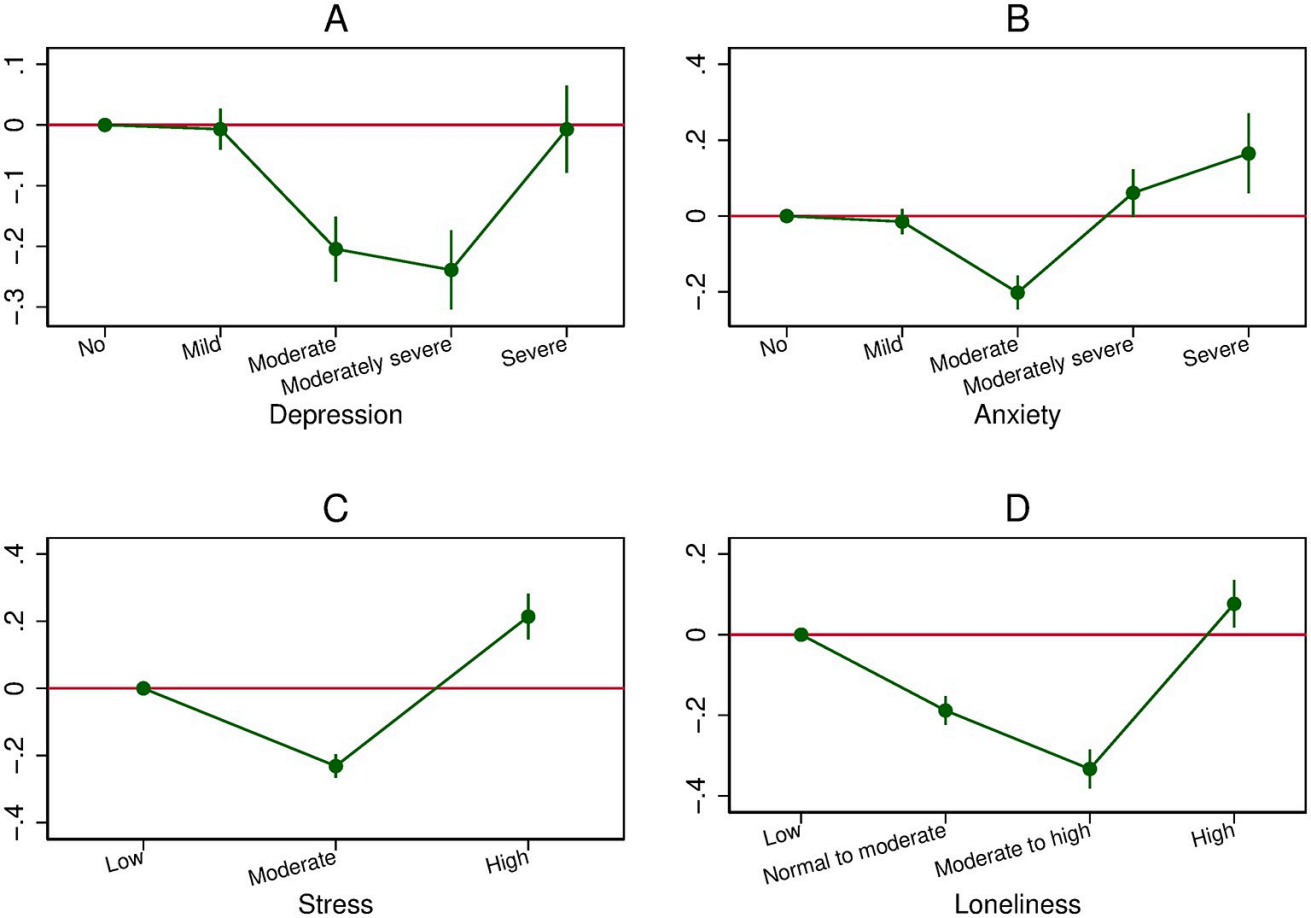
The U-shaped relationship between mental health and overall compliance (Pooled OLS model). A: Relationship between depression and overall compliance. B: Relationship between anxiety and overall compliance. C: Relationship between stress and overall compliance. D: Relationship between loneliness and overall compliance.

**Fig 3:**
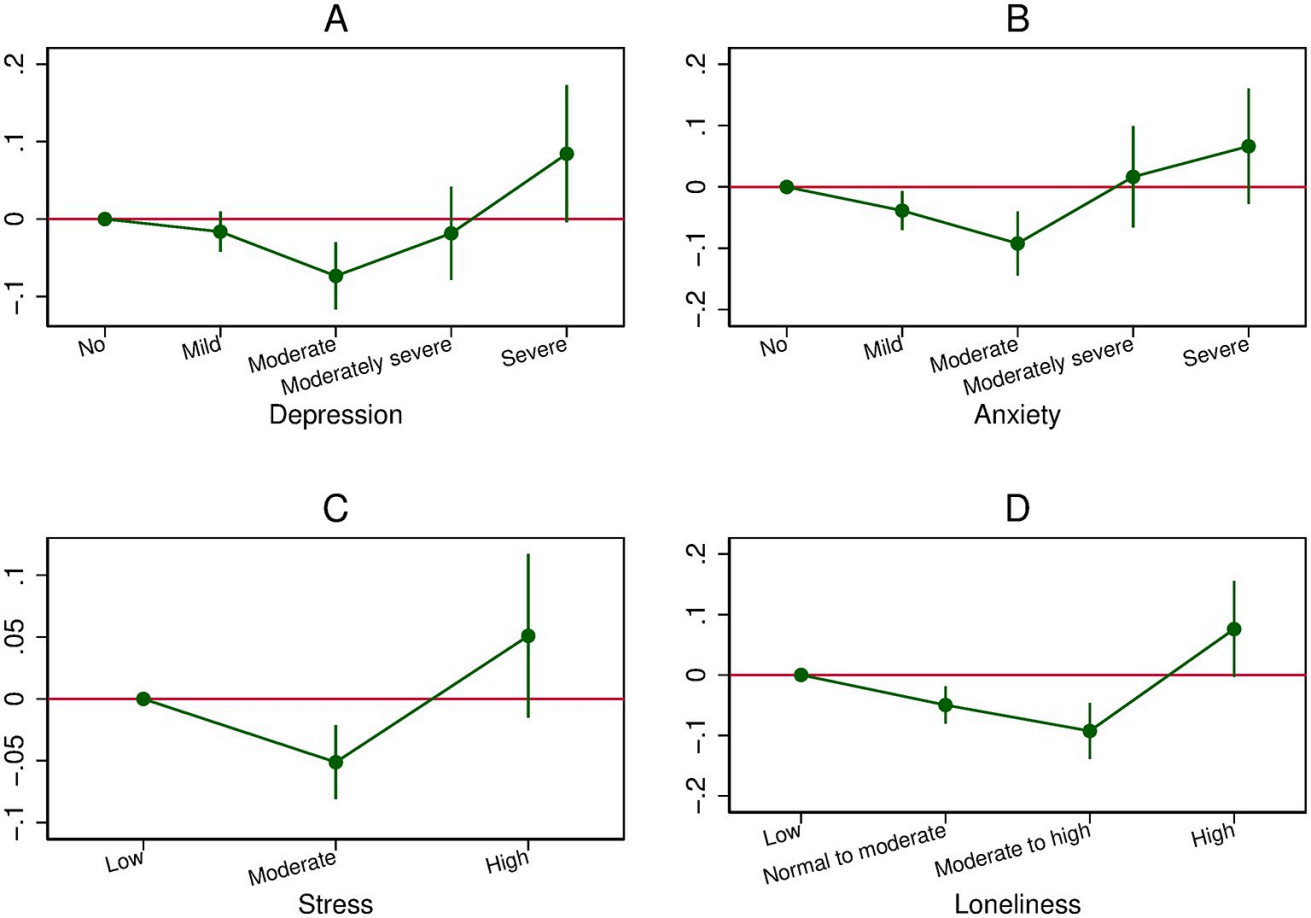
The U-shaped relationship between mental health and overall compliance (OLS-FE model). A: Relationship between depression and overall compliance. B: Relationship between anxiety and overall compliance. C: Relationship between stress and overall compliance. D: Relationship between loneliness and overall compliance.

**Fig 4:**
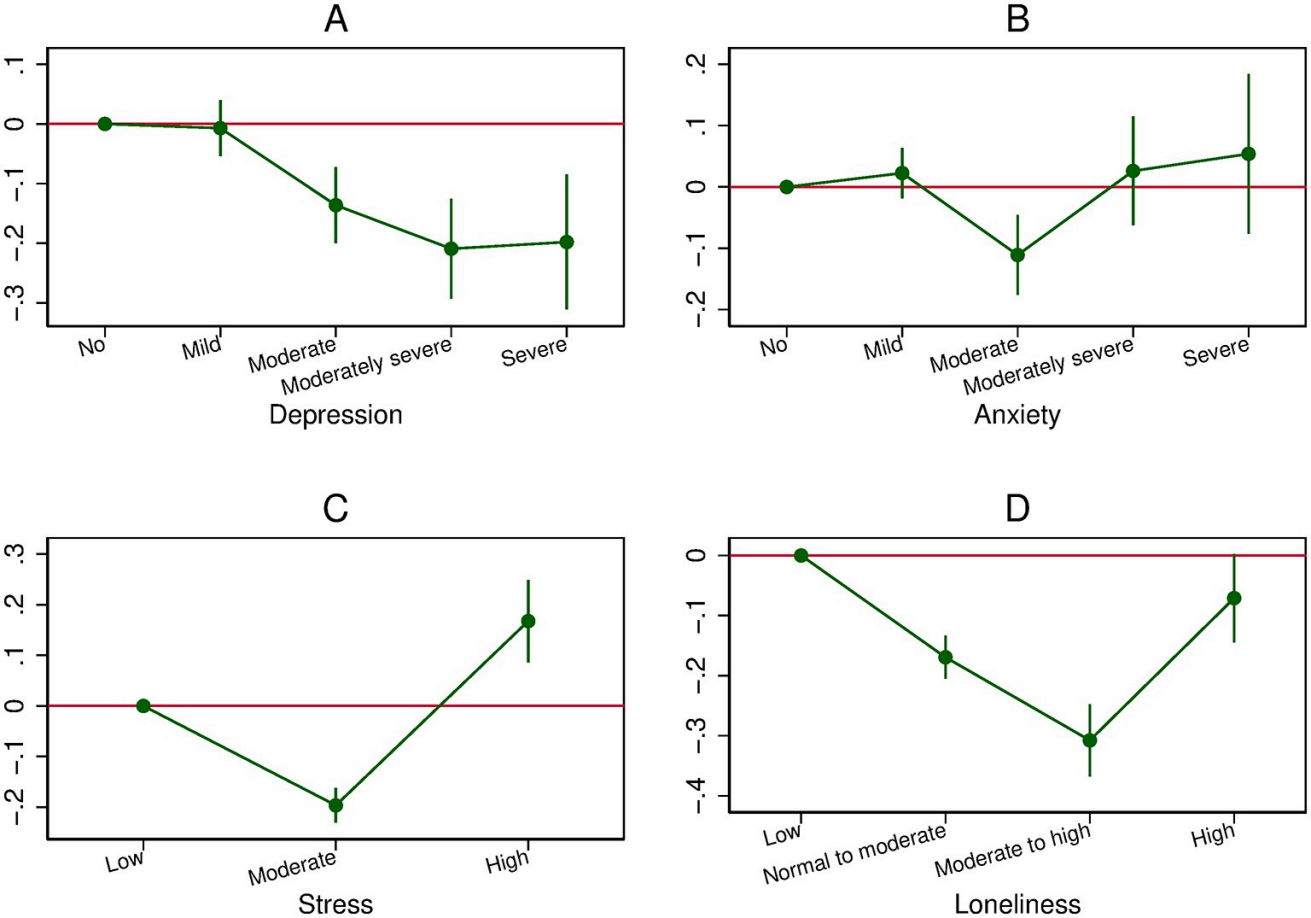
The relationship between mental health, measured in wave 1, and overall compliance, measured in waves 2 to 4 (Lag model). A: Relationship between depression and overall compliance. B: Relationship between anxiety and overall compliance. C: Relationship between stress and overall compliance. D: Relationship between loneliness and overall compliance.

In this section, we report our main estimation results showing that the association between mental health and overall compliance is U-shaped in the full sample. The estimates are shown graphically in Fig 2 (Pooled OLS model), Fig 3 (OLS-FE model), and Fig 4 (Lag model). In each figure, we present the coefficients on the different levels of depression, anxiety, stress, and loneliness, using the first category (i.e., no depression, no anxiety, low level of stress, or low level of loneliness) as the reference category, as well as the 95% coefficient confidence intervals. Whatever the models, we always find that the association between mental health and overall compliance is U-shaped, with one exception (the association with depression in the Lag model).

More precisely, in the Pooled OLS model for depression, the left-hand side of the curve is flat, meaning that individuals with mild depression follow the recommendations as much as individuals with no depression. The slope then becomes negative, indicating that those with moderate depression follow the recommendations less (by 0.20, i.e., 0.17 standard deviation of overall compliance) than those with no depression, and that those with severe depression follow the recommendations even less (by 0.24, i.e., 0.20 standard deviation). Finally, when the slope turns positive, we observe that individuals with severe depression follow the recommendations as much as those with no depression. A similar U shape is found for anxiety, stress, and loneliness.

In the OLS-FE model, we find a U shape for the four mental health indicators. In other words, transitions to low and high levels of mental health are associated with higher scores of overall compliance, while transitions to medium levels of mental health are associated with lower scores. The magnitude of the coefficients is generally smaller than in the Pooled OLS approach. The coefficient on mild depression is statistically similar to that of no depression; moderate depression is associated with a lower score of following recommendations by 0.07; moderately severe depression has the same effect as no depression; and severe depression is associated with a higher score of overall compliance by 0.08.

Yet, these OLS-FE results may be driven by a mixing of population groups that would be characterised both by different patterns of transitions between mental health states and by different links between mental health and overall compliance. In S1 File, we report additional OLS-FE results for samples restricted either to individuals who are in very good mental health at wave 1 (Fig B.1 in S1 File), or to individuals who are *not* in very good mental health at wave 1 (Fig B.2 in S1 File), or to individuals whose state does not improve over time (Fig B.3 in S1 File). We thus focus on sub-samples for which we can *a priori* assume more homogeneous patterns of transitions between mental health levels. We almost always still find U shapes for the four mental health constructs in these sub-samples, albeit to a lesser extent for depression and stress than for anxiety and loneliness. Hence, the U shape is not driven by some heterogeneity in the mental health/overall compliance relationship between groups of individuals characterised by different patterns of variations in mental health.

Finally, the Lag model, in which reverse causation is highly unlikely, shows U shapes for anxiety, stress, and loneliness, but a negative association with depression (Fig 4). Consequently, anxiety, stress, and loneliness levels at baseline have a U-shaped effect on overall compliance later, while the depression level at baseline has a detrimental impact on overall compliance later.

## Specific health behaviours

In the previous section, we showed a U-shaped association between mental health and overall compliance, in various specifications. We now turn to examine the role of specific health behaviours in this shape. These specific health behaviours are captured by the CBS and the ADH *items*.

If two conditions are fulfilled, specific health behaviours will play a role in the U-shaped association between mental health and overall compliance. These two conditions are the following: (1) the association between mental health and some specific behaviours is U-shaped, and (2) among the specific behaviours for which a U shape is found, some behaviours have a significant positive effect on overall compliance.

### Effect of specific health behaviours on overall compliance

As explained above, to drive the U shape, specific health behaviours must have a significant effect on overall compliance. This will be the case if individuals evaluate their overall tendency to follow recommendations from the implementation of some specific behaviours. To test this, we estimate the impact of specific health behaviours (as measured by the items) on overall compliance.

**Fig 5:**
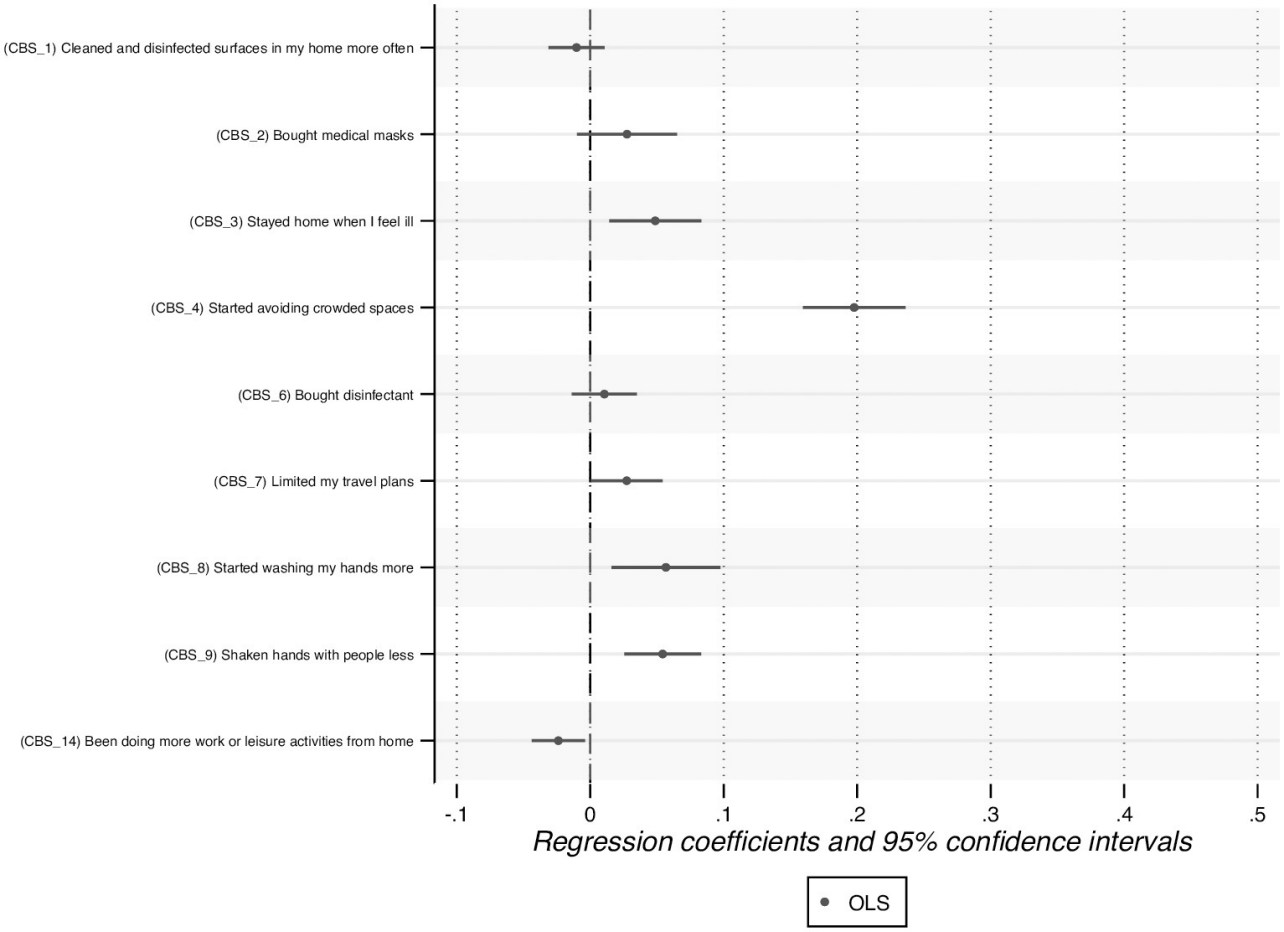
Association between CBS items and overall compliance.

We regress overall compliance on the nine CBS items, controls, and country fixed effects. Because CBS items are only measured in wave 1, the model is estimated using OLS and cannot be estimated using OLS-FE. The coefficients on the items are shown in Fig 5. In the regression, five CBS items have a positive and significant effect (at the 5% level) on the overall tendency to follow the recommendations: stayed home when I feel ill (CBS_3), started avoiding crowded spaces (CBS_4), limited my travel plans (CBS_7), started washing my hands more (CBS_8), and shaken hands with people less (CBS_9). The effect of CBS_4 (started avoiding crowded spaces) on overall compliance is especially large.

**Fig 6:**
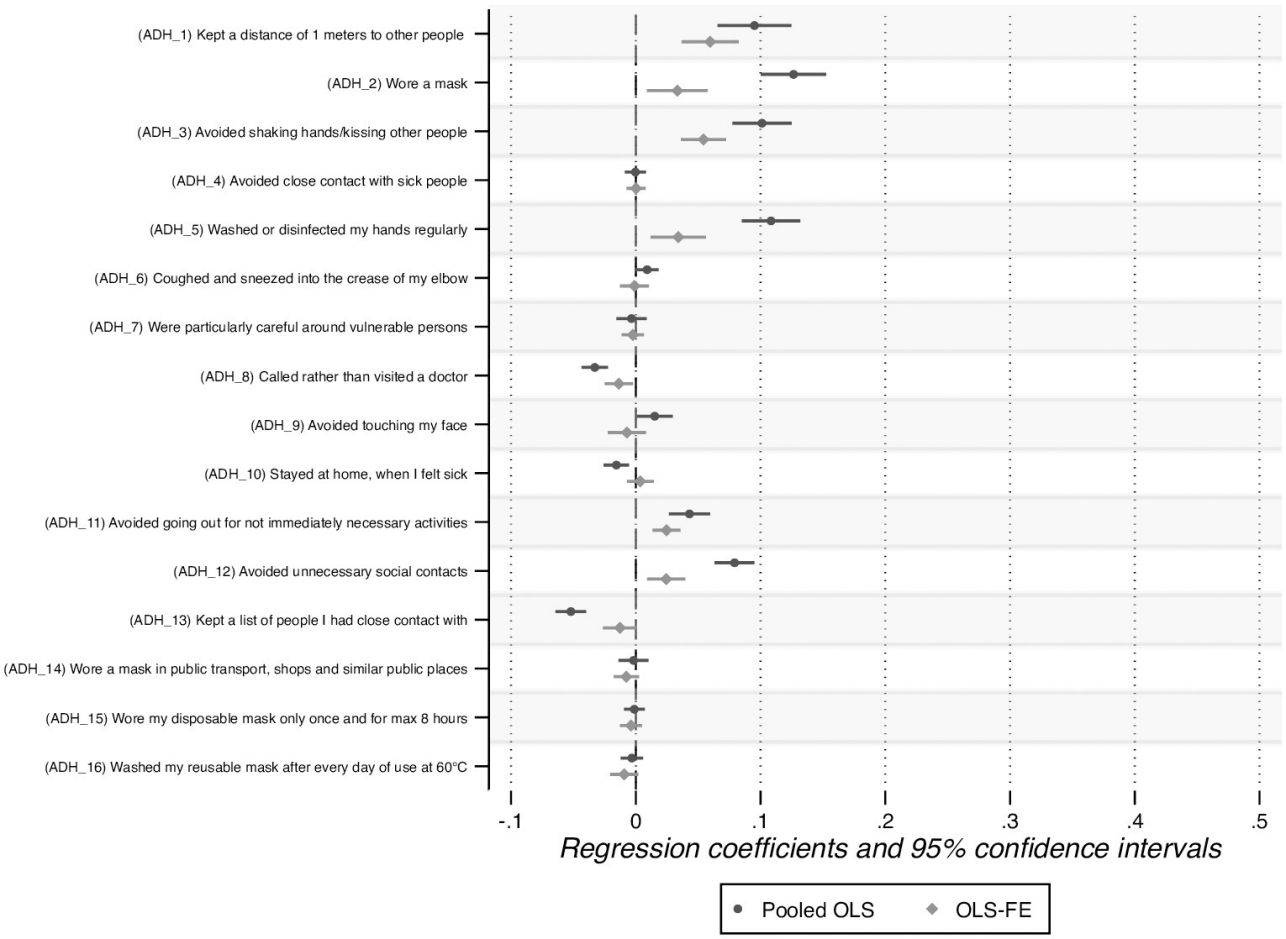
Association between ADH items and overall compliance.

The results of the analysis of ADH items are reported in Fig 6. Given that these items are assessed in waves 3 and 4, we are able to estimate an OLS-FE model in addition to the OLS model. In both the OLS and the OLS-FE models, six items have a positive and significant effect on overall compliance: kept a distance of 2 meters to other people (ADH_1), wore a mask when the distance of 2 meters could not be kept (ADH_2), avoided shaking hands with or kissing other people (ADH_3), washed or disinfected my hands regularly (ADH_5), avoided going out for not immediately necessary activities (ADH_11), and avoided unnecessary social contact (ADH_12). In addition, ADH_9 (avoided touching my face) has a significant effect in the OLS model, but not in the OLS-FE model.

We have thus established that individuals evaluate their overall tendency to follow recommendations from the implementation of 11 or 12 specific behaviours (i.e., five CBS items and six or seven ADH items). We demonstrate below that the association between mental health and some of these 11–12 specific behaviours is U-shaped.

### The U-shaped association between mental health and a number of specific health behaviours

We examine whether the association between mental health and specific health behaviours is U-shaped, by re-estimating our main model using each CBS and ADH item as a dependent variable (Table 1).

**Table 1:** Specific health behaviours for which a U shape is found.

Model	Sample	Cases in which a U shape is observed	
		Explained variable	Explanatory variable
		Specific health behaviour	Mental health
OLS	Full sample, wave 1^1^	Avoiding crowded spaces (CBS_4)	Stress, loneliness
		Washing hands more (CBS_8)	Stress, loneliness
		Shaking hands less (CBS_9)	Depression, stress, loneliness
OLS-FE	Full sample, waves 3 and 4^2^	Avoiding shaking hands with or kissing other people (ADH_3)	Depression, stress, loneliness
		Washing or disinfecting hands regularly (ADH_5)	Stress
		Being particularly careful around vulnerable persons (ADH_7)	Stress
		Wearing disposable mask only once and for max. 8 hours (ADH_15)	Depression
Lag	Full sample, waves 3 and 4^2^	Kept a distance of 2 meters to other people (ADH_1)	Depression, stress, loneliness
		Wore a mask, when the distance of 2 meters could not be kept (ADH_2)	Depression, anxiety, stress, loneliness
		Avoiding shaking hands with or kissing other people (ADH_3)	Anxiety, stress, loneliness
		Washing or disinfecting hands regularly (ADH_5)	Depression, anxiety, stress, loneliness
		Coughed and sneezed into the crease of my elbow or a tissue (ADH_6)	Loneliness
		Were particularly careful around vulnerable persons (ADH_7)	Loneliness
		Avoided touching my face (ADH_9)	Depression, loneliness
		Avoided unnecessary social contacts (ADH_12)	Loneliness
		Wore a mask in public transport, shops, and similar public places (ADH_14)	Depression, stress, loneliness

^1^ Data on CBS items are available in wave 1 only.

^2^ Data on ADH items are available in waves 3 and 4.

When CBS items are the explained variables, we can only estimate the OLS model due to data limitations. We find a U-shaped association between mental health and three CBS items: avoiding crowded spaces (CBS_4), washing hands more (CBS_8), and shaking hands less (CBS_9). Importantly, these three behaviours positively influence the overall tendency to follow the recommendations, and CBS_4 is the CBS item which has the greatest effect on this overall tendency, as shown in Fig 5. Our analysis thus confirms that CBS items play a role in the U-shaped association between mental health and overall compliance.

We reach a similar conclusion for ADH items. In the OLS-FE model, a U shape is found for four ADH items (ADH_3, ADH_5, ADH_7, and ADH_15) (Table 1). Two items in this list (ADH_3 and ADH_5) have a positive and significant influence on overall compliance (see the OLS-FE model in Fig 6). Finally, in the Lag model, we observe a U shape for nine ADH items (ADH_1, ADH_2, ADH_3, ADH_5, ADH_6, ADH_7, ADH_9, ADH_12, and ADH_14) (Table 1). Of those, six items (ADH_1, ADH_2, ADH_3, ADH_5, ADH_9, and ADH_12) have a positive and significant impact on overall compliance (see the OLS model in Fig 6).

### The relationship between mental health and the CBS and ADH scores

Unlike for overall compliance and for specific behaviours, we do not often observe a U-shaped association for the CBS and ADH *scores*. In fact, we often (but not always) find that poorer mental health goes hand in hand with better health behaviours as measured by these scores. Given that the correlation between overall compliance and the scores is low, the difference in shapes between overall compliance (U shape) and the scores is not surprising. Details are provided in Tables C.1–C.3 and related comments in S1 File.

## Understanding the U shape

The U-shaped association between mental health and overall compliance may be produced by the aggregation of heterogeneous effects across distinct populations. We here specifically study heterogeneity by country and by gender.

We first estimate our OLS-FE and Lag models by country separately. The results, summarised in Table 2, show that the U shape is very commonly observed. In particular, in the OLS-FE model, a U shape is found for each of the four mental health indicators for France and for three mental health measures for Italy, while in the Lag model, a U-shape is found for three mental health variables for Germany, Italy, Spain, and Sweden. These results highlight that the U shape found in the sample containing all countries is not produced by mixing countries with negative relationships and countries with positive relationships.

**Table 2:** Relationship between mental health and overall compliance, by country (OLS-FE and Lag models).

Country	Model	Observations	Depression	Anxiety	Stress	Loneliness
France	OLS-FE	4920	U shape	U shape	U shape	U shape
	Lag	3346	Other	Other	U shape	U shape
Germany	OLS-FE	4928	Insignificant U shape	Insignificant	U shape	U shape
	Lag	3348	U shape	Insignificant U shape	U shape	U shape
Italy	OLS-FE	5042	U shape	U shape	Stability and increase	U shape
	Lag	3465	U shape	Insignificant	U shape	U shape
Spain	OLS-FE	4965	Insignificant	Insignificant	Insignificant	Insignificant U shape
	Lag	3361	Other	U shape	U shape	U shape
Sweden	OLS-FE	3182	Insignificant	U shape	Insignificant	Insignificant
	Lag	2046	U shape	Other	U shape	U shape

We also investigate heterogeneity by gender. In our data, females report a greater overall tendency to follow the recommendations than males: the average response is 6.17 for females, versus 6.00 for males (this difference is statistically significant at the 1% level), and the probability of being in the highest category of overall compliance is 7.77 point higher for females than for males (Fig D.1 in S1 File).

We examine the association between mental health and overall compliance by gender. To do this, in the case of depression, we first create 10 population groups: (1) males without depression, (2) males with mild depression, … (5) males with severe depression, (6) females without depression, … and (10) females with severe depression. We then regress overall compliance on a series of dummies for whether the individual belongs to each of the population groups. We estimate the OLS-FE and the Lag models, and males without depression and females without depression serve as reference categories. The usual control variables are included. Similar models are estimated for anxiety, stress, and loneliness.

**Fig 7:**
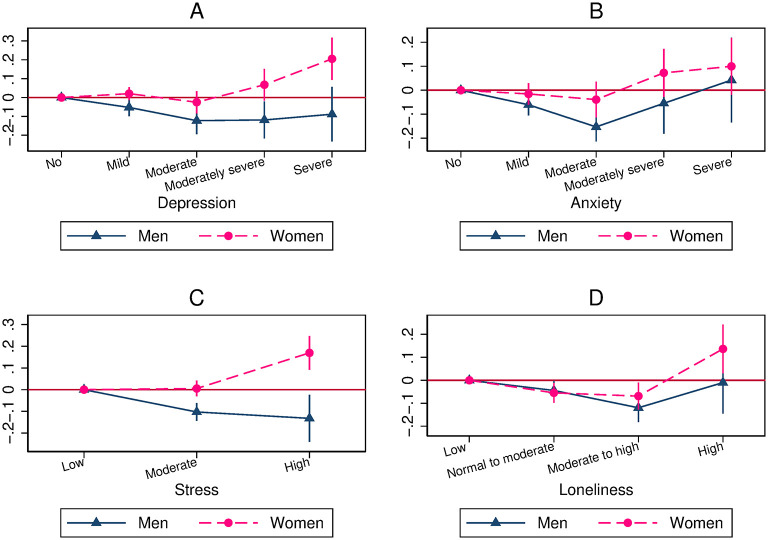
Relationship between mental health and overall compliance, by gender (OLS-FE model). A: Relationship between depression and overall compliance. B: Relationship between anxiety and overall compliance. C: Relationship between stress and overall compliance. D: Relationship between loneliness and overall compliance.

**Fig 8:**
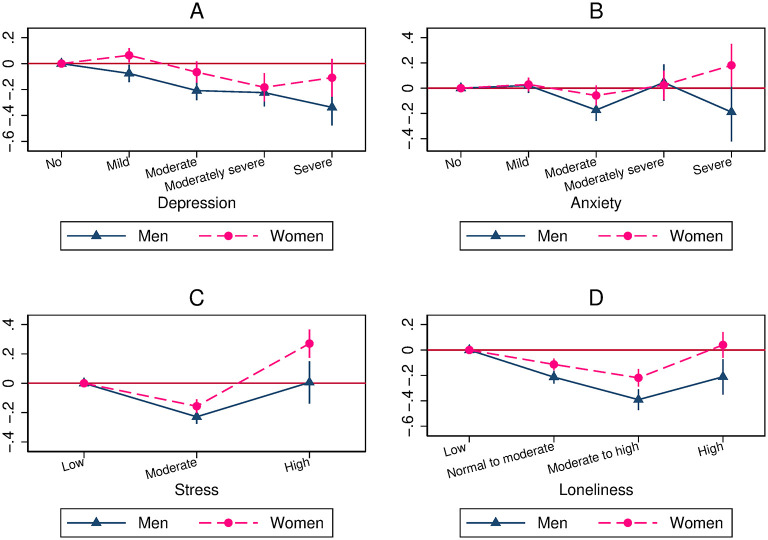
Relationship between mental health and overall compliance, by gender (Lag model). A: Relationship between depression and overall compliance. B: Relationship between anxiety and overall compliance. C: Relationship between stress and overall compliance. D: Relationship between loneliness and overall compliance.

Fig 7 (OLS-FE model) and Fig 8 (Lag model) show the coefficients on mental health categories for females and males. Results are consistent with a U shape for both genders for anxiety (OLS-FE model), stress (Lag model), and loneliness (OLS-FE and Lag models).

In the OLS-FE model for depression and stress, the U shape we found in the full sample containing females and males (Fig 3) is driven by a gender composition effect, with males contributing to the negative relationships found for low-levels of depression and stress and females contributing to the positive relationships found for the highest levels of these mental health measures (Fig 7). A question is then whether these differences in the association between mental health and health behaviour are driven by gender-specific psychological mechanisms.

In the Lag model for depression, the negative slope found in the full sample containing men and women (top-left subfigure in Fig 4) is due to a negative slope for both females and males (top-left subfigure in Fig 8). The absence of gender differences in the Lag model demonstrates that initial levels of depression (in wave 1) are similarly associated with overall compliance for men and women.

## Discussion

Using novel panel data on 7,766 individuals (22,878 observations) from five European countries during the COVID-19 pandemic, we investigate the relationship between mental health (as measured by depression, anxiety, stress, and loneliness) and the overall tendency to follow official recommendations regarding COVID-19 (i.e., overall compliance).

Our study is all the more relevant as the relationship between mental health and health behaviours is poorly understood. For instance, in some cases, a heightened state of anxiety leads individuals to adopt uncertainty-reducing actions, e.g., to voluntarily take more risks [5, 22]. In this context, anxiety will have a detrimental effect on (protective) health behaviours. However, individuals with more anxiety may also increase self-protection efforts. In that case, anxiety will have the opposite effect, since it will improve health behaviours. As also pointed out by Wright et al. [15], empirical findings on the relationship between mental health and (protective) health behaviours during the COVID pandemic are rather contradictory [9, 10, 15].

In contrast to the previous literature, we employ a flexible specification that allows the association between mental health and health behaviours to be non-monotonic. We find a U-shaped association between mental health and overall compliance: those with the lowest and highest levels of depression, anxiety, stress, and loneliness follow the recommendations more, while those with moderate levels of these variables follow them less, in the OLS model. Compared with poor and good mental health, moderate psychological problems thus decrease the overall ability to successfully engage in self-protection efforts. One possible interpretation is that moderate psychological problems decrease the motivation to comply. This result highlights the need for targeted interventions and support for individuals with moderate psychological problems. As far as we are aware, we are the first to report on this U shape (i.e., a negative followed by a positive association), which could explain some of the conflicting results highlighted in previous research (with either positive or negative associations) [9, 10, 15].

Moreover, for anxiety, stress, and loneliness, we also find U shapes when we estimate OLS-FE regressions (which account for individual unobserved characteristics that are fixed over time) and Lag models (in which we use mental health at baseline and in which reverse causation is highly unlikely). These results suggest that both the transitory component of the mental health measures (captured in the OLS-FE model) and their permanent components (captured in the Lag model) are associated with overall compliance in a U-shaped manner.

In contrast, results for depression depend on the choice of the model: while the OLS and OLS-FE regressions produce U-shaped associations, baseline depression in the Lag model displays a negative and monotonic relationship with overall compliance later. In other words, the higher his depression score at baseline, the less the individual follows official recommendations overall later. Higher depression scores at baseline may be related to overall resignation and fatalism [23], hence the belief that following recommendations is futile. Importantly, the results for depression highlight that the effect of a transitory depression state is not necessarily the same as that of the depression state at baseline. More generally, our findings show that we must be ready for the possibility that the relationship between depression (at baseline) and overall compliance is different from that between other mental health constructs (at baseline) and overall compliance.

After establishing that individuals evaluate their overall compliance from their implementation of a set of specific health behaviours (assessed by CBS and ADH items), we examine whether specific health behaviours are also U-shaped in mental health. For all models taken together, we find a U shape for thirteen specific health behaviours (three CBS items, in the OLS model, and 10 ADH items, in either the OLS-FE or the Lag model). The U shape for washing hands is very robust since it is found in all of our approaches. Our finding is different from some previous results: for instance, one study shows that anxiety reduces the odds of washing hands in Japan [9]. Taken together, our results highlight the role of specific health behaviours in the U shape in overall compliance. Interestingly, while baseline depression has a detrimental impact on overall compliance, it has a U-shaped effect on some specific health behaviours (e.g., wearing a mask and washing or disinfecting hands regularly).

Nevertheless, for many pairs of mental health measures and specific health behaviours, there is no U shape. This heterogeneity implies that in studies on the link between mental health and health behaviours, researchers need to be clear about what type of health behaviours they are talking about and be ready for the possibility that the effect of mental health is different for different health behaviours.

We also investigate cross-country heterogeneity. Our analysis does not provide evidence of important heterogeneity between the five countries, as U-shaped effects on overall compliance are found in the five countries. This similarity highlights the robustness of our main finding.

Previous research shows that even though men are more likely to die of COVID-19, they are less likely to see COVID-19 as a very serious health problem and are less compliant than women [24, 25]. However, regarding the link between mental health and overall compliance, our results are often similar for men and women. We find U shapes for both females and males in a number of specifications. Moreover, the detrimental effect of depression at baseline on overall compliance is found for both genders. In contrast, results for men and women are very different in some cases, generating gender composition effects. In particular, in the OLS-FE model, the U-shaped link between depression or stress and overall compliance in the full sample is driven by a gender composition effect, with males driving the negative slope for lower levels of depression and stress, and females driving the positive slope for higher levels of these mental health indicators.

Our study has several strengths. We exploit high quality data from the COME-HERE survey. Moreover, the longitudinal design allows us to increase causal leverage, by accounting for stable individual characteristics and by measuring mental health several weeks or months before assessing health behaviours. Most importantly, our econometric approach accounts for the fact that health behaviours may not monotonically increase or decrease when mental health deteriorates. This flexible method seems even more relevant as the previous literature provides mixed predictions and findings regarding the relationship between mental health and health behaviours.

Nevertheless, some limitations of the present study should be noted. First, we rely upon self-reported measures of health behaviours and mental health that may be prone to social desirability concerns. Findings on the existence of this social desirability bias are mixed [26, 27]. However, note that individual answers in the COME-HERE survey are confidential, which should decrease the social desirability bias. Second, while we attempt to approach causal inference by using the Lag and OLS-FE models, it remains possible that the omission of confounding factors could bias our results. By definition, confounding factors are variables that are correlated with both mental health and health behaviours. Future research could delve into this aspect, by testing if the results are robust to the inclusion of potential omitted variables. Finally, our study does not explore mediating and moderating effects (e.g., the roles of emotional states [28] and feelings of threat and self-efficacy [29]).

## Conclusion

Despite its limitations, the current study provides a detailed exploration of the relationship between four mental health indicators and a number of health behaviours, in five countries. As far as we are aware, this is the first study that highlights a U-shaped association between a set of mental health measures on the one hand and overall compliance and several specific health behaviours on the other hand. This finding may help reconcile contradictory results from the previous literature. As the pandemic runs its course, it may continue to put a strain on the psychological health of many people. Our results suggest that transitioning to an intermediate level of depression, anxiety, stress, or loneliness may lead to a decrease in overall compliance with public health recommendations. While a growing body of evidence emphasises that worsening psychological health may lead to an increase in behaviours that amplify the COVID-related risks [30–33], our analysis demonstrates that even moderate mental health problems can have detrimental effects on health behaviour. This highlights the importance of maintaining and expanding budgets for mental health policy.

## Supporting information

S1 File (PDF)
