# Supplementary material for: Mental health and the overall tendency to follow official recommendations against COVID-19: A U-shaped relationship?
Source: PLoS One. 2024 Jun 25;19(6):e0305833. doi: 10.1371/journal.pone.0305833 (PMC11198746; doi:10.1371/journal.pone.0305833)
Supplement: S1 File — (PDF) [file pone.0305833.s001.pdf]

Mental health and the overall tendency to follow official recommendations against COVID-19: A U-shaped relationship?

Supporting information

Bénédicte Apouey, Rémi Yin, Fabrice Etilé, Alan Piper, Claus Vögele

Appendix A: Method

Background on recommendations in the five countries over time

To combat the COVID-19 pandemic, governments and policymakers have made many behaviours and practices recommended and, in some cases, mandatory. Many of these recommendations for the citizens of our five countries (and elsewhere) have been consistent (between waves 1 and 4 of the COME-HERE survey). For example, the good hygiene practice behaviours of washing and disinfecting hands, not touching one's face, and social distancing have been perennial. Other measures have differed both between the five countries we study and over time during the pandemic. As is well known, Sweden adopted a different approach to combating the pandemic, with the chief epidemiologist stating as late as August 2020 that wearing masks might be counterproductive (Milne and Khan 2020) – a stance in stark contrast to the approach in the four other countries investigated. This is highlighted in Figure A.1, which shows changes over time during the pandemic in official recommendations regarding wearing a mask, staying at home, protecting the elderly, and restrictions on gathering, in the five countries. The data comes from Oxford University's Blavatnik School of Government COVID-19 Government Response Tracker (Hale et al. 2021). The horizontal axis indicates the dates, including the dates of the waves of the COME-HERE data. The vertical axis shows the strength of the recommendation (the higher the number, the greater the strength; what each specific figure indicates is explained in Table A.1). The figure shows some variety in these measures over time, whereas other recommendations are perennial (washing and disinfecting hands; avoiding touching one's face; and social distancing).

Figure A.1: Strength of government recommendations for wearing a mask, staying at home, protecting the elderly, and restrictions on gathering, in the five countries during the pandemic

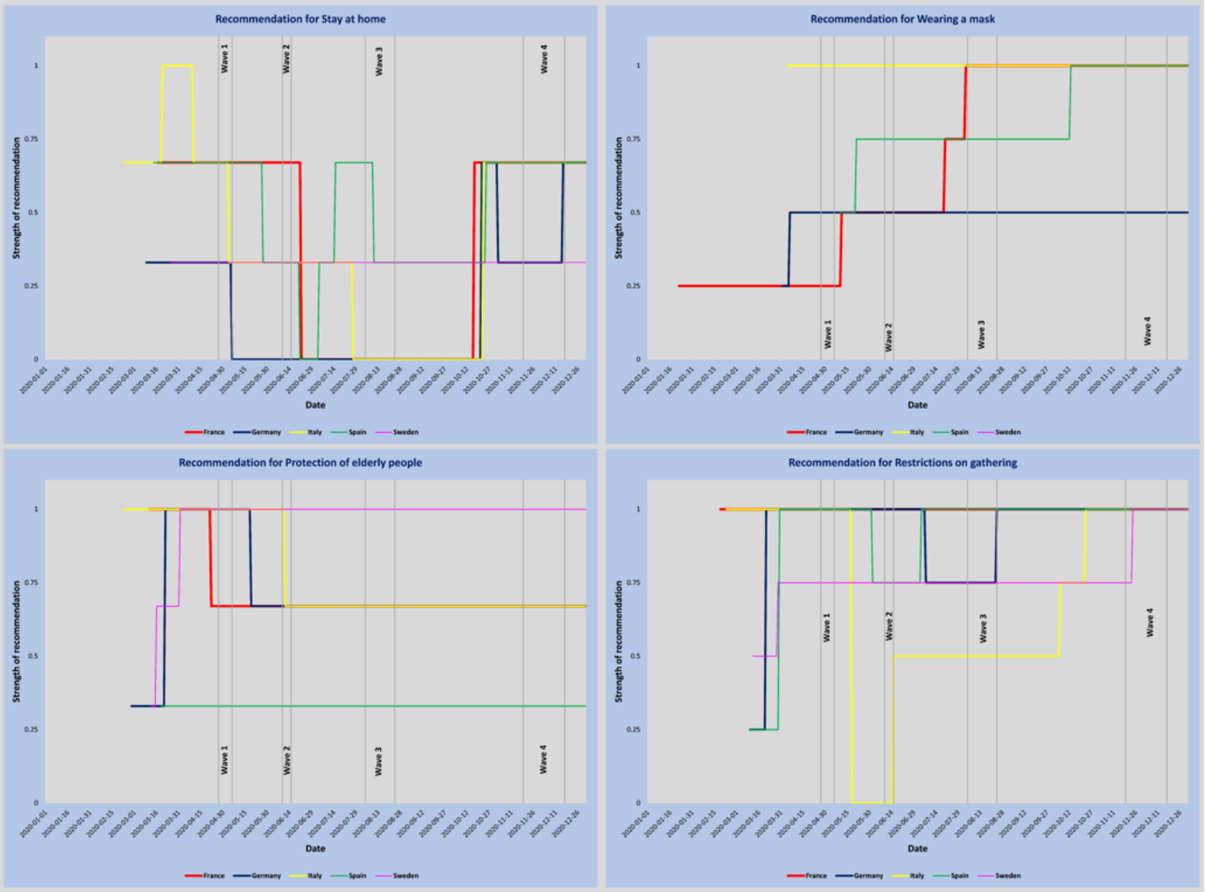

Source: Figure created based on data from the COVID-19 Government Response Tracker. See Hale et al. (2021) and https://www.bsg.ox.ac.uk/research/research-projects/covid-19-government-response-tracker.
Notes: Waves indicate when individuals were surveyed in the COME-HERE panel dataset.

Table A.1: COVID-19 strength of recommendations from Figure A.1

	**Facial coverings**
0	No policy
0.25	Recommended
0.5	Required in some specified shared / public spaces outside the home with other people present, or some situations when social distancing not possible
0.75	Required in all shared / public spaces outside the home with other people present, or all situations when social distancing not possible
1	Required outside the home at all times regardless of location or presence of other people
	**Protection of elderly people**
	Note: LTCF stands for long term care facility.
0	No measures
0.33	Recommended isolation, hygiene, and visitor restriction measures in LTCFs and / or elderly people to stay at home
0.67	Narrow restrictions for isolation, hygiene in LTCFs, some limitations on external visitors and / or restrictions protecting elderly people at home
1	Extensive restrictions for isolation and hygiene in LTCFs, all non-essential external visitors prohibited, and / or all elderly people required to stay at home and not leave the home with minimal exceptions, and receive no external visitors
	**Stay at home measures**
0	No measures
0.33	Recommend not leaving house
0.67	Require not leaving house with exceptions for daily exercise, grocery shopping, and "essential" trips
1	Require not leaving house with minimal exceptions (e.g., allowed to leave once a week, or only one person can leave at a time, etc.)
	**Restrictions on gathering**
0	No restrictions
0.25	Restrictions on very large gatherings (above 1,000 people)
0.5	Restrictions on gatherings between 101–1,000 people
0.75	Restrictions on gatherings between 11–100 people
1	Restrictions on gatherings of 10 people or less

References

Hale, Thomas, Noam Angrist, Rafael Goldszmidt, Beatriz Kira, Anna Petherick, Toby Phillips, Samuel Webster, Emily Cameron-Blake, Laura Hallas, Saptarshi Majumdar, and Helen Talow. 2021. "A global panel database of pandemic policies (Oxford COVID-19 Government Response Tracker)." *Nature Human Behaviour* 5(4): 529–538.

Milne, Robert, and Mehreen Khan. 2020. “Coronavirus outlier Sweden chooses its own path on face masks.” *The Financial Times*.

Appendix A (cont.): The internal consistency of the Coronavirus Behavior Scale for reasonable behaviours

Table A.2: Cronbach's alpha of the Coronavirus Behavior Scale (CBS) for reasonable behaviours

Item	(1) Obs	(2) Sign	(3) Item-test correlation	(4) Item-rest correlation	(5) Average inter-item covariance	(6) Alpha
CBS_1	7999	+	0.69	0.58	0.44	0.80
CBS_2	7999	+	0.65	0.51	0.45	0.80
CBS_3	7999	+	0.64	0.51	0.46	0.80
CBS_4	7999	+	0.71	0.63	0.47	0.79
CBS_6	7999	+	0.73	0.61	0.43	0.79
CBS_7	7999	+	0.63	0.51	0.47	0.80
CBS_8	7999	+	0.70	0.61	0.47	0.79
CBS_9	7999	+	0.55	0.43	0.50	0.81
CBS_14	7999	+	0.51	0.36	0.50	0.82
Test scale					0.47	0.82

Source: Wave 1 of the COME-HERE (COVID-19, MEntal HEalth, REsilience and Self-Regulation) survey.

Appendix A (cont.): Construction of the three adherence scores

In waves 3 and 4, respondents are asked 16 questions capturing adherence (see the Method section). In these 16 items, we recode the responses in the following way: (0) Never or Not applicable; (1) Almost never; (2) Sometimes; (3) Fairly often; (4) Very often; and (5) All the time.

Using the principal component analysis that we present below, we are able to construct three aggregate scores of adherence (Score adh A, Score adh B, and Score adh C) by summing the relevant items.

We first study internal consistency using Cronbach's alpha. This constitutes a widely used psychometric tool to study the correlation of items within a scale and therefore to know whether the items measure the same construct. Cronbach's alpha equals 0 for independent items and 1 for perfectly correlated items. A high Cronbach's alpha is evidence that item responses are driven by the same latent theoretical construct. It is also possible to investigate the contribution of a given item to a scale by looking at the variation of the Cronbach's alpha when we remove the item. Table A.3 presents the results. The overall Cronbach's alpha is 0.85, indicating good internal consistency.

Table A.4 presents the results of the exploratory factor analysis for adherence to test the validity of the questionnaire. We rely on a principal component method with varimax rotation using the polychoric correlation matrix of items. The scree plot in Figure A.2 of the principal component method leads to retain three factors. Items loading more than 0.5 on a factor are kept to create a score for the factor. The first score, Score adh A, seems to correspond to the most explicit recommended measures (ADH_1, ADH_3, ADH_4, ADH_5, ADH_6, ADH_7, ADH_11, ADH_12). The second score, Score adh B, represents more implicit measures (ADH_8, ADH_10, ADH_13). Finally, the third score, Score adh C, represents items related to mask wearing (ADH_2, ADH_14, ADH_15, ADH_16). The Kaiser-Meyer-Olkin index for the complete model is equal to 0.89, which is excellent (Kaiser 1974). Almost all items load higher than 0.5 to a factor. ADH_9 does not display a loading factor higher than 0.5. For this reason, we remove ADH_9 to construct the scores of adherence in the current analysis.

Table A.3: Cronbach's alpha of adherence

Item	(1) Obs	(2) Sign	(3) Item-test correlation	(4) Item-rest correlation	(5) Average inter-item covariance	(6) Alpha
ADH_1	10979	+	0.53	0.47	0.89	0.85
ADH_2	10979	+	0.59	0.51	0.85	0.84
ADH_3	10979	+	0.48	0.42	0.89	0.85
ADH_4	10979	+	0.55	0.44	0.84	0.85
ADH_5	10979	+	0.53	0.48	0.89	0.85
ADH_6	10979	+	0.50	0.40	0.87	0.85
ADH_7	10979	+	0.60	0.51	0.84	0.84
ADH_8	10979	+	0.58	0.48	0.83	0.84
ADH_9	10979	+	0.67	0.61	0.83	0.84
ADH_10	10979	+	0.54	0.44	0.84	0.85
ADH_11	10979	+	0.65	0.58	0.83	0.84
ADH_12	10979	+	0.62	0.56	0.85	0.84
ADH_13	10979	+	0.55	0.46	0.85	0.84
ADH_14	10979	+	0.49	0.39	0.86	0.85
ADH_15	10979	+	0.61	0.51	0.82	0.84
ADH_16	10979	+	0.56	0.47	0.83	0.84
Test scale					0.85	0.85

Source: Waves 3 and 4 of the COME-HERE (COVID-19, MEntal HEalth, REsilience and Self-Regulation) survey.

Table A.4: Exploratory factor analysis

Variable	Label	Factor 1	Factor 2	Factor 3	Uniqueness	SMC	KMO
ADH_1	Kept a distance of two meters to other people	0.64			0.37	0.47	0.91
ADH_2	Wore a mask			0.86	0.05	0.74	0.83
ADH_3	Avoided shaking hands/kissing other people	0.81			0.13	0.68	0.88
ADH_4	Avoided close contact with sick people	0.61			0.30	0.49	0.89
ADH_5	Washed or disinfected my hands regularly	0.74			0.22	0.60	0.92
ADH_6	Coughed and sneezed into the crease of my elbow	0.52			0.47	0.37	0.92
ADH_7	Were particularly careful around vulnerable persons	0.67			0.28	0.54	0.93
ADH_8	Called rather than visited a doctor		0.85		0.18	0.67	0.86
ADH_9	Avoided touching my face				0.35	0.52	0.94
ADH_10	Stayed at home, when I felt sick		0.86		0.14	0.67	0.83
ADH_11	Avoided going out for not immediately necessary activities	0.56			0.17	0.66	0.86
ADH_12	Avoided unnecessary social contacts	0.70			0.16	0.68	0.87
ADH_13	Kept a list of people I had close contact with		0.85		0.17	0.65	0.86
ADH_14	Wore a mask in public transport, shops, and similar public places			0.81	0.24	0.60	0.82
ADH_15	Wore my disposable mask only once and for max 8 hours			0.60	0.39	0.50	0.93
ADH_16	Washed my reusable mask after every day of use at 60°C			0.51	0.45	0.44	0.93
							0.89

Notes: This table presents the main component analysis (MCA) using polychoric correlations. The analysis is performed with varimax rotation and we specify a one factor solution.
SMC: Squared multiple correlation.
KMO: Kaiser-Meyer-Olkin index.

Figure A.2: Scree plot

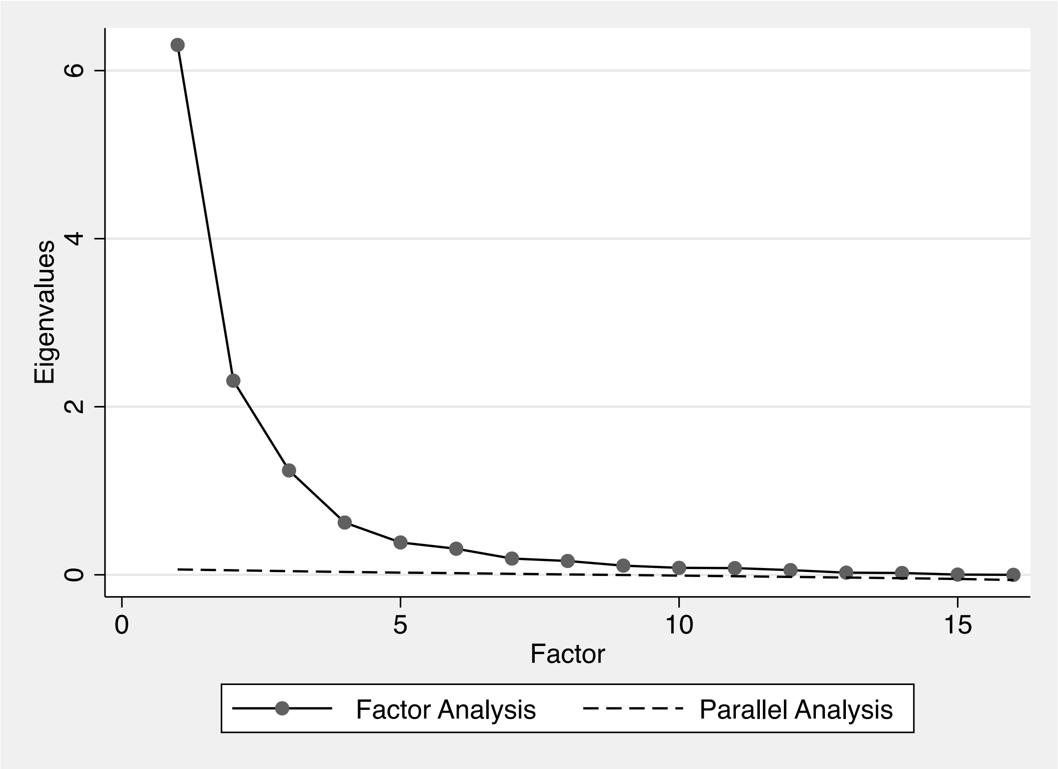

Notes: This graph plots the eigenvalues of the factor analysis without restricting the number of factors. The dashed line is generated by a random correlation matrix with the same numbers of observations and variables as the original data.

References

Kaiser, Henry F. 1974. “An index of factorial simplicity.” *Psychometrika* 39(1): 31-36.

Appendix A (cont.): Internal consistency and descriptive statistics for mental health

Table A.5: Cronbach's alpha of depression (PHQ-9)

Item	(1) Obs	(2) Sign	(3) Item-test correlation	(4) Item-rest correlation	(5) Average inter-item covariance	(6) Alpha
PHQ9_1	22878	+	0.80	0.74	0.44	0.92
PHQ9_2	22878	+	0.85	0.80	0.43	0.91
PHQ9_3	22878	+	0.75	0.67	0.43	0.92
PHQ9_4	22878	+	0.79	0.72	0.43	0.92
PHQ9_5	22878	+	0.80	0.74	0.43	0.92
PHQ9_6	22878	+	0.83	0.77	0.43	0.92
PHQ9_7	22878	+	0.83	0.78	0.43	0.92
PHQ9_8	22878	+	0.77	0.71	0.45	0.92
PHQ9_9	22878	+	0.75	0.68	0.45	0.92
Test scale					0.44	0.93

Source: Waves 1 to 4 of the COME-HERE (COVID-19, MEntal HEalth, REsilience and Self-Regulation) survey.

Table A.6: Cronbach's alpha of anxiety (GAD-7)

Item	(1) Obs	(2) Sign	(3) Item-test correlation	(4) Item-rest correlation	(5) Average inter-item covariance	(6) Alpha
GAD7_1	22878	+	0.86	0.81	0.52	0.93
GAD7_2	22878	+	0.87	0.82	0.52	0.92
GAD7_3	22878	+	0.88	0.83	0.52	0.92
GAD7_4	22878	+	0.87	0.81	0.52	0.93
GAD7_5	22878	+	0.81	0.74	0.55	0.93
GAD7_6	22878	+	0.82	0.76	0.54	0.93
GAD7_7	22878	+	0.85	0.78	0.53	0.93
Test scale					0.53	0.94

Source: Waves 1 to 4 of the COME-HERE (COVID-19, MEntal HEalth, REsilience and Self-Regulation) survey.

Table A.7: Cronbach's alpha of stress (PSS)

Item	(1)<br>Obs	(2)<br>Sign	(3)<br>Item-test correlation	(4)<br>Item-rest correlation	(5)<br>Average inter-item covariance	(6)<br>Alpha
PSS_1	22878	+	0.64	0.54	0.45	0.79
PSS_2	22878	+	0.73	0.64	0.42	0.78
PSS_3	22878	+	0.74	0.65	0.41	0.78
PSS_4	22878	-	0.45	0.30	0.48	0.82
PSS_5	22878	-	0.52	0.39	0.47	0.81
PSS_6	22878	+	0.63	0.52	0.44	0.79
PSS_7	22878	-	0.42	0.26	0.49	0.82
PSS_8	22878	-	0.58	0.46	0.45	0.80
PSS_9	22878	+	0.68	0.57	0.43	0.79
PSS_10	22878	+	0.74	0.66	0.41	0.78
Test scale					0.44	0.81

Source: Waves 1 to 4 of the COME-HERE (COVID-19, MEntal HEalth, REsilience and Self-Regulation) survey.

Table A.8: Cronbach's alpha of loneliness (ULS-8)

Item	(1)<br>Obs	(2)<br>Sign	(3)<br>Item-test correlation	(4)<br>Item-rest correlation	(5)<br>Average inter-item covariance	(6)<br>Alpha
ULS8_1	22878	+	0.70	0.57	0.34	0.79
ULS8_2	22878	+	0.79	0.70	0.32	0.77
ULS8_3	22878	-	0.40	0.23	0.42	0.83
ULS8_4	22878	+	0.81	0.73	0.32	0.77
ULS8_5	22878	+	0.79	0.70	0.32	0.77
ULS8_6	22878	-	0.46	0.28	0.40	0.83
ULS8_7	22878	+	0.61	0.48	0.37	0.80
ULS8_8	22878	+	0.74	0.63	0.33	0.78
Test scale					0.35	0.82

Source: Waves 1 to 4 of the COME-HERE (COVID-19, MEntal HEalth, REsilience and Self-Regulation) survey.

Table A.9: Descriptive statistics for mental health variables

	Mean	Std. Dev.	Min	Max
Depression – PHQ-9 score	6.16	6.17	0	27
Anxiety – GAD-7 score	5.43	5.26	0	21
Stress – PSS score	15.29	7.39	0	40
Loneliness – ULS-8 score	16.23	5.26	8	32
Observations	22878			

Figure A.3: Histogram of depression (PHQ-9)

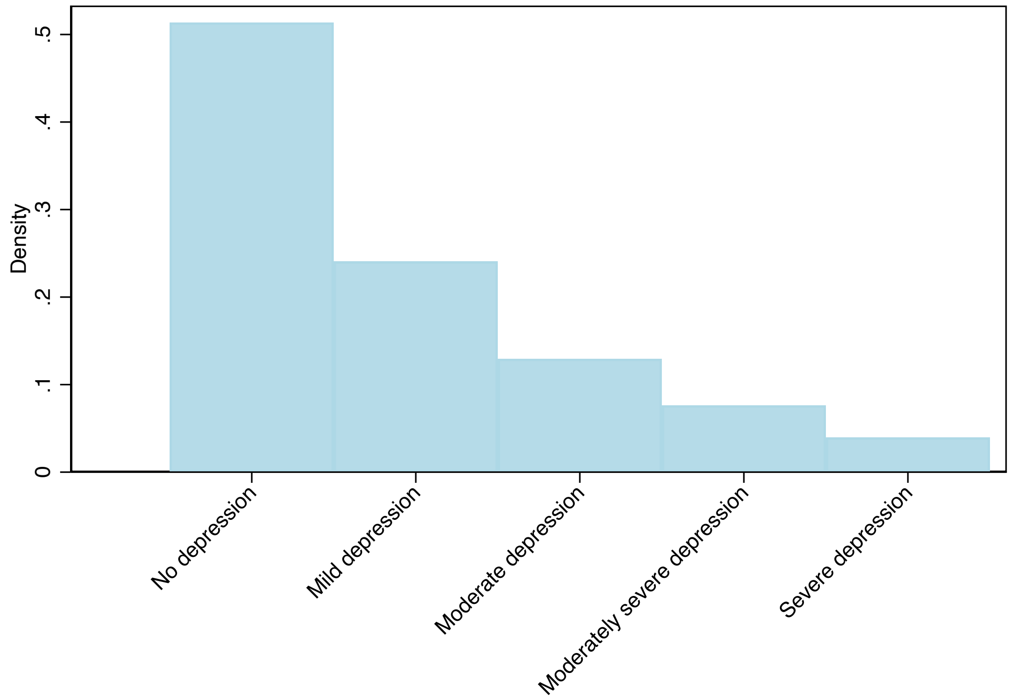

Figure A.4: Histogram of anxiety (GAD-7)

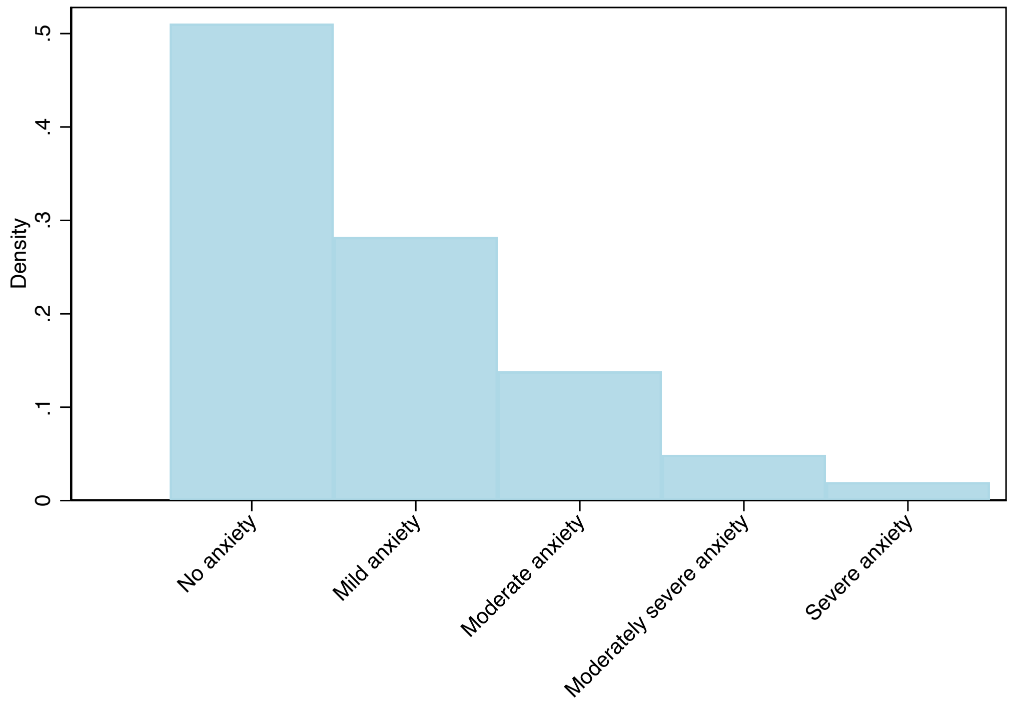

Figure A.5: Histogram of stress (PSS)

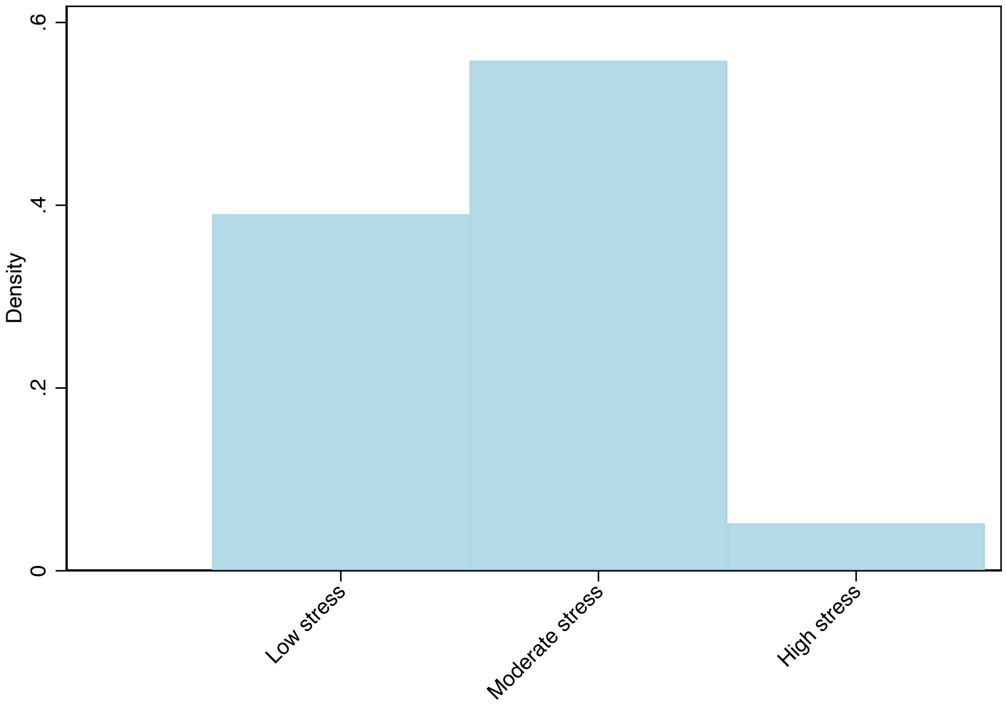

Figure A.6: Histogram of loneliness (ULS-8)

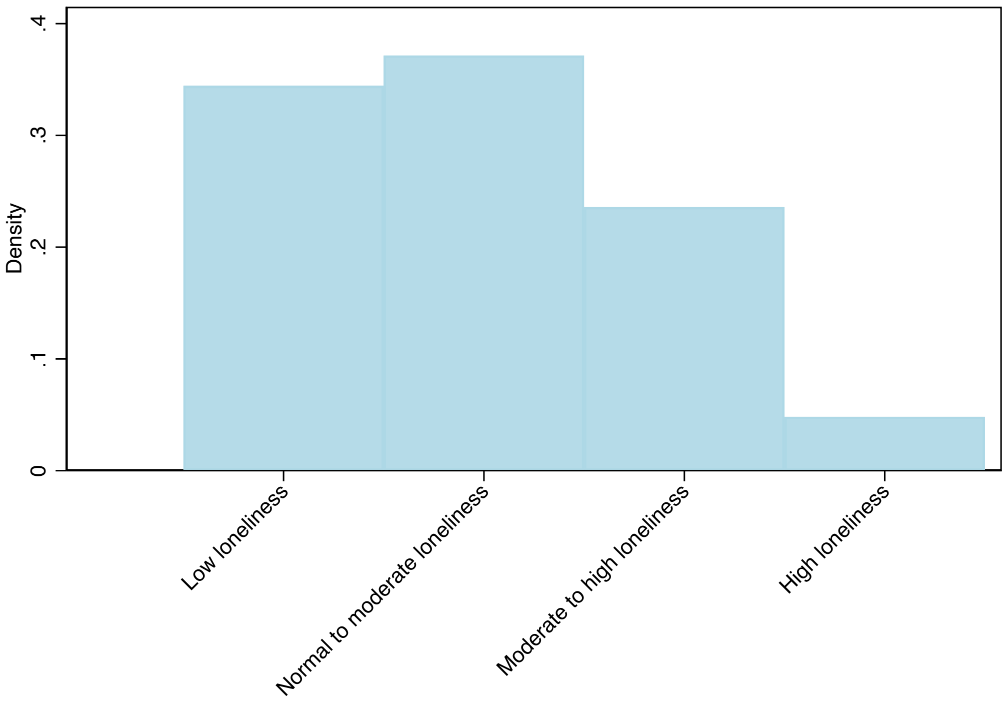

68

Appendix A (cont.): Correlation between mental health indicators

Table A.10: Correlation matrix between mental health variables

	Depression score (PHQ-9)	Anxiety score (GAD-7)	Stress score (PSS)	Loneliness score (ULS-8)
Depression score (PHQ-9)	1			
Anxiety score (GAD-7)	0.83***	1		
Stress score (PSS)	0.67***	0.69***	1	
Loneliness score (ULS-8)	0.62***	0.55***	0.64***	1

Source: Waves 1 to 4 of the COME-HERE (COVID-19, MEntal HEalth, REsilience and Self-Regulation) survey.
Notes: *** p<0.01; ** p<0.05; * p<0.1.

Appendix A (cont.): Control variables

In the regression models, we include a set of individual-, household-, and aggregate-level controls. We have good reason to believe that these controls may have an effect on health behaviours, and including these controls can help prevent an omitted variable bias from affecting our results.

Individual- and household-level controls

We use information on gender, age, marital status, household composition, education, working from home, income, income change, confidence in the government and in health services, and knowledge of the disease.

Demographic characteristics

Gender is coded as a dummy.

Regarding age, we create a series of dummies capturing the age groups (less than 24, 25–34, 35–44, 45–54, 55–64, and 65+).

Marital status is coded in four categories (single, never married; single, divorced or widowed; in a relationship / married but living apart; in a relationship / married and cohabiting).

To capture household composition, we use the number of children and the number of individuals aged 65+ living in the household. We also include a dummy for when we do not know whether there are older adults in the household.

Socioeconomic status

Socioeconomic status is captured by education level, whether the individual works at home, income, and income change.

The education indicator has three categories: low (i.e., primary education; general education (secondary) school), medium (O-levels or equivalent; A-levels or equivalent; vocational education / training) and high education level (master's degree; PhD; MD; etc.).

Job status captures whether the individual works at home, does not work at home, or does not work.

Information on household income is given in brackets (0–1,250 Euros; 1,250–2,000 Euros; 2,000–4,000; 4,000–6,000 Euros; 6,000–8,000 Euros; 8,000–12,500 Euros; >12,500 Euros). We use the middle of the brackets and compute equivalised income by household size. We then standardise income by country. We also create a dummy capturing missing values.

In addition, we employ two dummies for whether individual income decreased or decreased since the previous wave.

Confidence

We use two scales capturing the level of confidence in the government and in health services. The questions are the following: "How much confidence do you have that the government of your country of residence can handle COVID-19 well?" [scale from 1 ("None at all") to 7 ("Full confidence")] and "How much confidence do you have that the health service of your current country of residence can cope during COVID-19 ?" [scale from 1 ("No confidence") to 7 ("Full confidence")]. We standardise these scales by country.

Knowledge of the disease

Knowledge of the disease is measured using the following question: "How would you rate your knowledge level on COVID-19?" with responses ranging from 1 ("Very poor knowledge") to 7 ("Very good knowledge"). We standardise the variable by country.

Aggregate-level controls

We combine our COME-HERE data with aggregate data from the Oxford University's Blavatnik School of Government COVID-19 Government Response Tracker (Hale et al. 2021). Important for our analysis is the Tracker's stringency index that records how strict the lockdown type policies are, that restrict the behaviour of people in a country. This is calculated using all of the tracker's containment and closure policy indicators and an indicator recording public information campaigns (which are as follows: school and university closures; workplace

closures; the cancelling of public events; restrictions on public gatherings; public transport restrictions; stay at home orders; restrictions on internal movement; restrictions on foreign travel; and evidence about coordinated public information campaigns).

In addition to the stringency index, we also use information on the number of daily confirmed deaths due to COVID-19 the day before the interview in each country of interest. Economic models of rational epidemics have demonstrated the importance of endogenous variations in self-protective behaviours in epidemic dynamics (Geoffard and Philipson 1996; Toxvaerd 2020). Self-protection efforts react endogenously to the epidemics, so that an increase in the incidence rate is likely to increase self-protection efforts.

References

Geoffard, Pierre-Yves, and Tomas Philipson. 1996. "Rational epidemics and their public control." *International Economic Review* 37 (3): 603–624.

Hale, Thomas, Noam Angrist, Rafael Goldszmidt, Beatriz Kira, Anna Petherick, Toby Phillips, Samuel Webster, Emily Cameron-Blake, Laura Hallas, Saptarshi Majumdar, and Helen Talow. 2021. "A global panel database of pandemic policies (Oxford COVID-19 Government Response Tracker)." *Nature Human Behaviour* 5(4): 529–538.

Toxvaerd, Flavio M. O. 2020. "Equilibrium social distancing." *Working Papers in Economics*, University of Cambridge.

Appendix B: The U-shaped relationship between mental health and overall compliance

Results for population groups defined using mental health level at baseline or mental health evolution over time

This appendix reports additional regression results to investigate whether the U shape is driven by some heterogeneity in the mental health / compliance relationship between individuals heterogeneous in their baseline mental health and / or in their observed transitions across mental health states. For instance, the negative slope of the U shape (on the left) may be produced by individuals always oscillating between medium and excellent levels of mental health, while the positive slope may correspond to individuals always oscillating between poor and medium levels of mental health. Hence, the mixing of these two populations characterised by correlations of opposite signs between mental health and overall compliance would eventually explain the U shape.

To investigate this explanation, we first keep waves 1 and 2 only and re-estimate the OLS-FE model restricting the sample to individuals who are in very good health in wave 1. Here, the association of interest is identified only by worsenings of mental health indicators between wave 1 and 2 for individuals who are in very good mental health at baseline. Results of the OLS-FE model are provided in Figure B.1. For the top-left figure, the sample contains individuals with no depression in wave 1, while for the top-right figure, it contains individuals with no anxiety in wave 1. The samples of the two bottom figures are defined in a similar way. We still find a U shape for anxiety and loneliness, but the associations remain negative for transitions to severe depression or stress states. However, the confidence intervals are large, as we observe a small number of such transitions.

In Figure B.2, we restrict the sample to individuals who are *not* in very good mental health in wave 1. In the top-left figure, the sample contains individuals with mild to severe depression in wave 1. In the top-right figure, it contains individuals with mild to severe anxiety in wave 1. The samples of the remaining figures are defined accordingly. We find a U shape for our four

mental health indicators.

Last, we also re-estimate our model restricting the sample to individuals whose mental health remains stable or worsens over the four waves. Individuals whose health improves at some point are thus deleted from the sample from the moment this improvement occurs. The results, reported in Figure B.3, are consistent with a U shape (except for depression for which the effect is nonmonotonic but not U-shaped).

Taken together, these figures are rather supportive of the existence of a U-shaped relationship, independently of the patterns of transitions across mental health states that may characterise specific groups of the population.

Figure B.1: Relationship between mental health and overall compliance in waves 1 and 2, for individuals in excellent mental health in wave 1 (OLS-FE model)

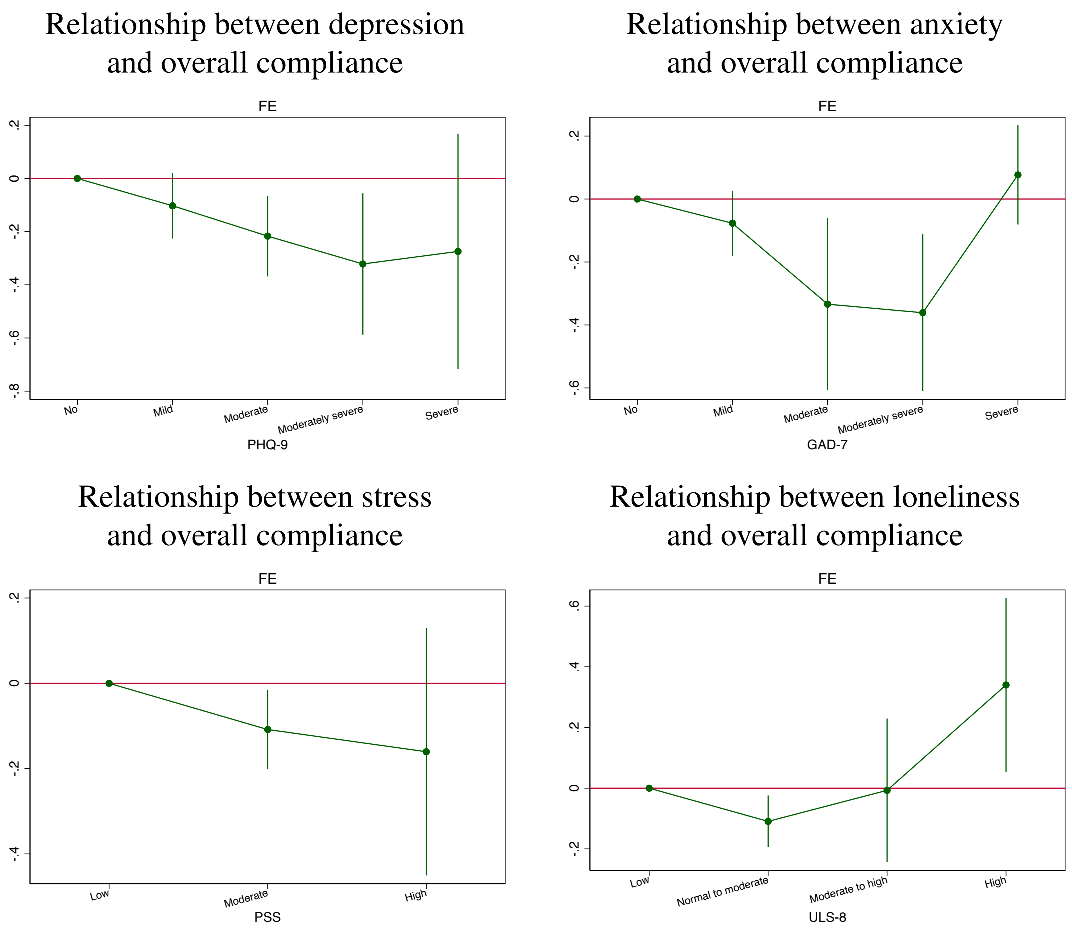

Source: COME-HERE (COVID-19, MEntal HEalth, REsilience and Self-Regulation) survey.
Notes: The samples contain observations from waves 1 and 2, for individuals who, in wave 1, have no depression (for the top-left figure), no anxiety (for the top-right figure), a low stress level (for the bottom-left figure), and a low loneliness level (for the bottom-right figure). The graphs present the effects of psychological health on overall compliance.

Figure B.2: Relationship between mental health and overall compliance in waves 1 and 2, for individuals who do *not* report excellent mental health in wave 1 (OLS-FE model)

Relationship between depression and overall compliance

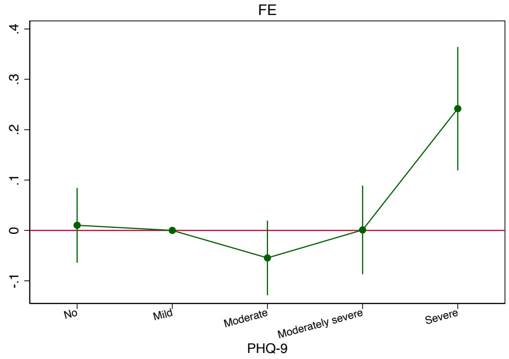

Relationship between anxiety and overall compliance

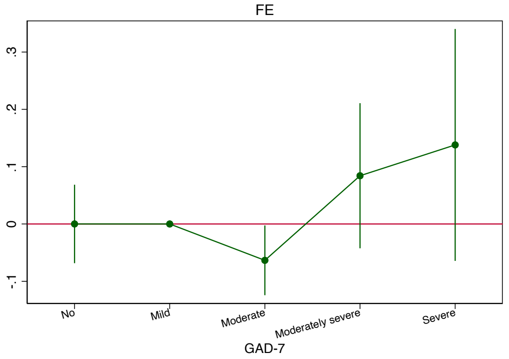

Relationship between stress and overall compliance

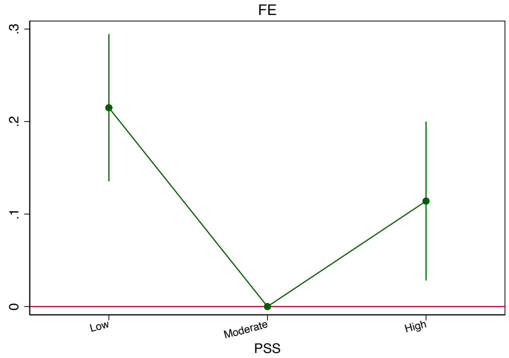

Relationship between loneliness and overall compliance

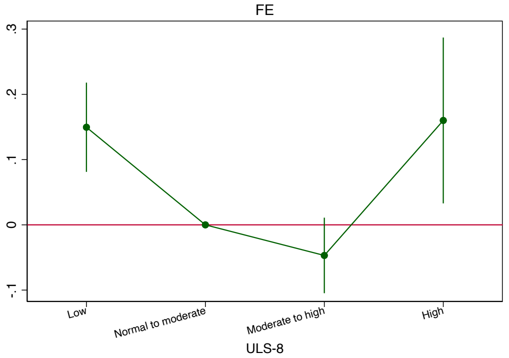

Source: COME-HERE (COVID-19, MEntal HEalth, REsilience and Self-Regulation) survey.
Notes: The samples contain observations from waves 1 and 2, for individuals who, in wave 1, have mild to severe depression (for the top-left figure), mild to severe anxiety (for the top-right figure), moderate or high stress (for the bottom-left figure), and normal-to-moderate to high loneliness (for the bottom-right figure). The graphs present the effects of psychological health on overall compliance.

Figure B.3: Relationship between mental health and overall compliance, for the sample of individuals whose mental health deteriorates or remains the same over time (OLS-FE model)

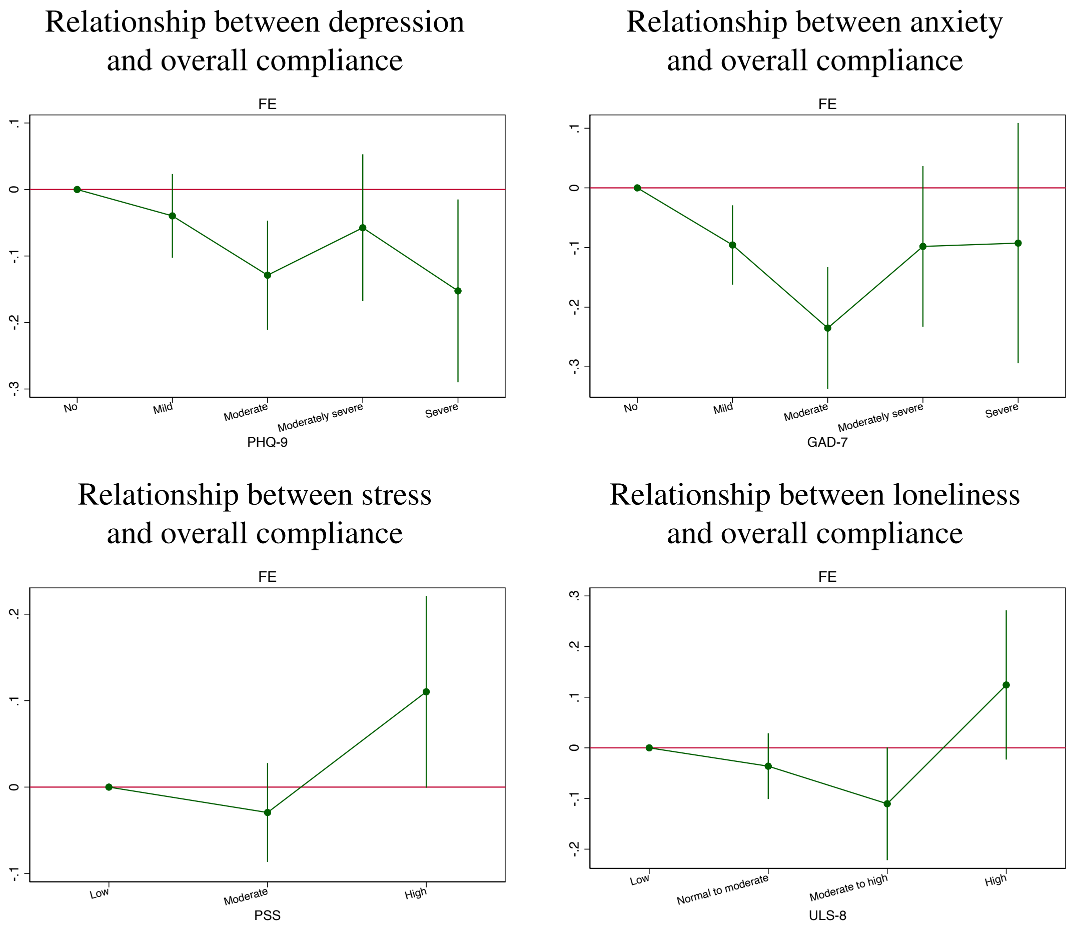

Source: COME-HERE (COVID-19, MEntal HEalth, REsilience and Self-Regulation) survey.
Notes: The sample contains observations of individuals whose health remains the same or deteriorates over time. An individual is dropped from the sample from the wave in which her health improves. The graphs present the effects of psychological health on overall compliance.

Appendix C: Specific health behaviours

Relationship between mental health and the CBS and adherence scores

Unlike for overall compliance and for specific behaviours, we do not often observe a U-shaped association for the CBS and adherence *scores*. In fact, we often (but not always) find that poorer psychological health goes hand in hand with better health behaviours as measured by these scores.

More precisely, when we regress the CBS score (for reasonable behaviours) on psychological health (Table C.1 (OLS)), we observe that higher levels of anxiety or stress are associated with a higher CBS score. However, the relationship between depression and the CBS score is nonmonotonic but not U-shaped, and the link between loneliness and the CBS score is not significant.

Regarding the adherence scores, results often indicate that poorer psychological health is positively associated with higher adherence (Tables C.2 (OLS-FE model) and C.3 (Lag model)). In the OLS-FE models, this is the case for nine models out of 12. In the Lag models, results are less clear since this is the case in only five models out of 12. Note that the Lag model shows that loneliness has a U-shaped effect on two adherence scores (Scores adh A and C).

The difference in shapes between overall compliance (U shape) and the scores is not surprising, given that the correlation between overall compliance and the scores is low (the correlation between overall compliance and the CBS score equals 0.34, while the correlation between overall compliance and the three adherence scores equals 0.43, 0.04, and 0.28).

Table C.1: Relationship between mental health and the CBS score (reasonable), in wave 1 (OLS model)

	(1)<br>CBS score<br>(reasonable)	(2)<br>CBS score<br>(reasonable)	(3)<br>CBS score<br>(reasonable)	(4)<br>CBS score<br>(reasonable)
Mild depression	0.985***			
	(0.198)			
Moderate depression	0.276			
	(0.236)			
Moderately severe depression	1.015***			
	(0.329)			
Severe depression	2.784***			
	(0.401)			
Mild anxiety		1.590***		
		(0.222)		
Moderate anxiety		1.545***		
		(0.248)		
Moderately severe anxiety		3.021***		
		(0.294)		
Severe anxiety		4.494***		
		(0.415)		
Moderate stress			0.559**	
			(0.225)	
High stress			1.376***	
			(0.339)	
Normal to moderate loneliness				0.177
				(0.236)
Moderate to high loneliness				0.132
				(0.398)
High loneliness				0.578
				(0.429)
Model	OLS	OLS	OLS	OLS
Obs	7312	7312	7312	7312

Source: Wave 1 of the COME-HERE (COVID-19, MEntal HEalth, REsilience and Self-Regulation) survey.
Notes: This table presents the results of the regression of the CBS score on depression categories in column (1), anxiety categories in column (2), stress categories in column (3), and loneliness categories in column (4). The reference category is no depression in column (1), no anxiety in column (2), low stress level in column (3), and low loneliness level in column (4). We include control variables and country fixed effects. Standard errors are clustered at the date of interview and country level.
*** p<0.01; ** p<0.05; * p<0.1.

Table C.2: Relationship between mental health and the three adherence scores, in waves 3 and 4 (OLS-FE model)

	(1) Score adh A	(2) Score adh A	(3) Score adh A	(4) Score adh A	(5) Score adh B	(6) Score adh B	(7) Score adh B	(8) Score adh B	(9) Score adh C	(10) Score adh C	(11) Score adh C	(12) Score adh C
Mild depression	0.057**				0.085***				-0.003			
	(0.023)				(0.021)				(0.022)			
Moderate depression	0.011				0.132***				0.008			
	(0.037)				(0.035)				(0.034)			
Moderately severe depression	0.070				0.211***				0.062			
	(0.044)				(0.039)				(0.044)			
Severe depression	0.328***				0.417***				0.217***			
	(0.053)				(0.056)				(0.046)			
Mild anxiety		0.042*				0.052**				0.023		
		(0.025)				(0.026)				(0.017)		
Moderate anxiety		0.073*				0.146***				0.063*		
		(0.043)				(0.040)				(0.036)		
Moderately severe anxiety		0.151***				0.166***				0.069		
		(0.049)				(0.051)				(0.045)		
Severe anxiety		0.499***				0.418***				0.286***		
		(0.096)				(0.079)				(0.066)		
Moderate stress			-0.001				0.107***				0.013	
			(0.018)				(0.025)				(0.023)	
High stress			0.162***				0.108***				0.063	
			(0.047)				(0.042)				(0.042)	
Normal to moderate loneliness				0.048**				0.120***				0.015
				(0.021)				(0.020)				(0.020)
Moderate to high loneliness				0.061**				0.190***				0.086**
				(0.031)				(0.030)				(0.034)
High loneliness				0.092**				0.116**				0.089*
				(0.044)				(0.050)				(0.053)
Model	OLS-FE	OLS-FE	OLS-FE	OLS-FE	OLS-FE	OLS-FE	OLS-FE	OLS-FE	OLS-FE	OLS-FE	OLS-FE	OLS-FE
Country-wave fixed effects	Yes	Yes	Yes	Yes	Yes	Yes	Yes	Yes	Yes	Yes	Yes	Yes
Obs	10874	10874	10874	10874	10874	10874	10874	10874	10874	10874	10874	10874

Source: Waves 3 and 4 of the COME-HERE (COVID-19, MEntal HEalth, REsilience and Self-Regulation) survey.
Notes: The explained variable is score adh A in columns (1) to (4), score adh B in columns (5) to (8), and score adh C in columns (9) to (12). The adherence scores are standardised (for the full sample). The explanatory variable are depression categories in columns (1), (5), and (9), anxiety categories in columns (2), (6), and (10), stress categories in columns (3), (7), and (11), and loneliness categories in columns (4), (8), and (12). The reference categories are no depression, no anxiety, low stress level, and low loneliness level. We include control variables and country-wave and individual fixed effects. Standard errors are clustered at the date of interview and country level.
*** p<0.01; ** p<0.05; * p<0.1.

Table C.3: Relationship between lagged mental health and the three adherence scores, in waves 3 and 4 (Lag model)

	(1) score adh A	(2) score adh A	(3) score adh A	(4) score adh A	(5) score adh B	(6) score adh B	(7) score adh B	(8) score adh B	(9) score adh C	(10) score adh C	(11) score adh C	(12) score adh C
Mild depression	0.519**				0.891***				0.053			
	(0.227)				(0.120)				(0.100)			
Moderate depression	-0.124				1.686***				0.159			
	(0.268)				(0.158)				(0.166)			
Moderately severe depression	0.073				2.432***				-0.041			
	(0.346)				(0.198)				(0.194)			
Severe depression	1.092*				3.381***				0.568**			
	(0.574)				(0.315)				(0.223)			
Mild anxiety		0.815***				1.177***				0.373***		
		(0.182)				(0.140)				(0.117)		
Moderate anxiety		0.459				2.005***				0.297*		
		(0.289)				(0.139)				(0.162)		
Moderately severe anxiety		1.459***				2.658***				0.603***		
		(0.412)				(0.217)				(0.181)		
Severe anxiety		2.625***				3.127***				0.884***		
		(0.543)				(0.475)				(0.318)		
Moderate stress			0.003				1.773***				0.128	
			(0.175)				(0.099)				(0.104)	
High stress			1.122***				1.069***				-0.008	
			(0.289)				(0.203)				(0.165)	
Normal to moderate loneliness				-0.687***				0.665***				-0.274***
				(0.140)				(0.119)				(0.105)
Moderate to high loneliness				-1.444***				1.322***				-0.427***
				(0.281)				(0.125)				(0.149)
High loneliness				0.843**				1.366***				-0.185
				(0.388)				(0.264)				(0.228)
Model	Lag	Lag	Lag	Lag	Lag	Lag	Lag	Lag	Lag	Lag	Lag	Lag
Country-Wave	Yes	Yes	Yes	Yes	Yes	Yes	Yes	Yes	Yes	Yes	Yes	Yes
Obs	10874	10874	10874	10874	10874	10874	10874	10874	10874	10874	10874	10874

Source: Waves 3 and 4 of the COME-HERE (COVID-19, MEntal HEalth, REsilience and Self-Regulation) survey.
Notes: The explained variable is score adh A in columns (1) to (4), score adh B in columns (5) to (8), and score adh C in columns (9) to (12). The adherence scores are standardised (for the full sample). The explanatory variable are depression categories in columns (1), (5), and (9), anxiety categories in columns (2), (6), and (10), stress categories in columns (3), (7), and (11), and loneliness categories in columns (4), (8), and (12). The reference categories are no depression, no anxiety, low stress level, and low loneliness level. We include control variables and country-wave fixed effects. Standard errors are clustered at the date of interview and country level.
*** p<0.01; ** p<0.05; * p<0.1.

Appendix D: Understanding the U shape

Overall compliance by gender

Figure D.1: Histogram for overall compliance by gender

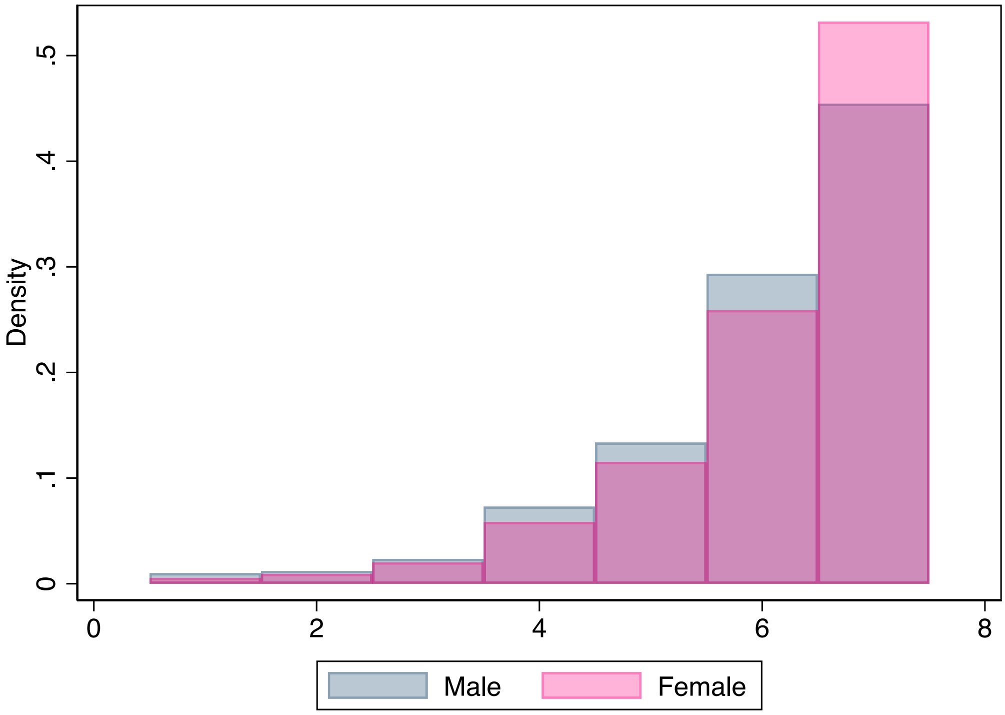

Source: Waves 1 to 4 of the COME-HERE (COVID-19, MEntal HEalth, REsilience and Self-Regulation) survey.
